# Identification of a New Equid Herpesvirus 1 DNA Polymerase (ORF30) Genotype with the Isolation of a C_2254_/H_752_ Strain in French Horses Showing no Major Impact on the Strain Behaviour

**DOI:** 10.3390/v12101160

**Published:** 2020-10-13

**Authors:** Gabrielle Sutton, Côme Thieulent, Christine Fortier, Erika S. Hue, Christel Marcillaud-Pitel, Alexis Pléau, Alain Deslis, Edouard Guitton, Romain Paillot, Stéphane Pronost

**Affiliations:** 1 LABÉO Frank Duncombe, 14280 Saint-Contest, France; gabrielle.sutton@laboratoire-labeo.fr (G.S.); come.thieulent@laboratoire-labeo.fr (C.T.); christine.fortier@laboratoire-labeo.fr (C.F.); erika.hue@laboratoire-labeo.fr (E.S.H.); romain.paillot@writtle.ac.uk (R.P.); 2 BIOTARGEN, Normandie Univ, UNICAEN, 14000 Caen, France; 3 ImpedanCELL, Normandie Univ, UNICAEN, 14280 Saint-Contest, France; 4 RESPE, 14280 Saint-Contest, France; c.marcillaud-pitel@respe.net; 5 INRAE, UE-1277 Plateforme d’Infectiologie Expérimentale (PFIE), Centre de Recherche Val de Loire, 37380 Nouzilly, France; alexis.pleau@inrae.fr (A.P.); alain.deslis@inrae.fr (A.D.); edouard.guitton@inrae.fr (E.G.); 6 School of Equine and Veterinary Physiotherapy, Writtle University College, Lordship Road, Writtle, Chelmsford CM1 3RR, UK

**Keywords:** equid herpesvirus 1, DNA polymerase, ORF30, EHV-1, antivirals, experimental infection

## Abstract

Equid herpesvirus 1 is one of the most common viral pathogens in the horse population and is associated with respiratory disease, abortion and still-birth, neonatal death and neurological disease. A single point mutation in the DNA polymerase gene (ORF30: A2254G, N752D) has been widely associated with neuropathogenicity of strains, although this association has not been exclusive. This study describes the fortuitous isolation of a strain carrying a new genotype C_2254_ (H_752_) from an outbreak in France that lasted several weeks in 2018 and involved 82 horses, two of which showed neurological signs of disease. The strain was characterised as U_L_ clade 10 using the equid herpesvirus 1 (EHV-1) multi-locus sequence typing (MLST) classification but has not been identified or isolated since 2018. The retrospective screening of EHV-1 strains collected between 2016 and 2018 did not reveal the presence of the C_2254_ mutation. When cultured in vitro, the C_2254_ EHV-1 strain induced a typical EHV-1 syncytium and cytopathic effect but no significant difference was observed when compared with A_2254_ and G_2254_ EHV-1 strains. An experimental infection was carried out on four Welsh mountain ponies to confirm the infectious nature of the C_2254_ strain. A rapid onset of marked respiratory disease lasting at least 2 weeks, with significant virus shedding and cell-associated viraemia, was observed. Finally, an in vitro antiviral assay using impedance measurement and viral load quantification was performed with three antiviral molecules (ganciclovir (GCV), aciclovir (ACV) and aphidicolin (APD)) on the newly isolated C_2254_ strain and two other A/G_2254_ field strains. The three strains showed similar sensitivity to ganciclovir and aphidicolin but both C_2254_ and A_2254_ strains were more sensitive to aciclovir than the G_2254_ strain, based on viral load measurement.

## 1. Introduction

Equid herpesvirus 1 (EHV-1) is an alphaherpesvirus, classified among the Herpesviridae family and the *Varicellovirus* genus [1]. EHV-1 is one of the most important viral pathogens infecting horses. Some cases have also been reported in other equid and non-equid mammals [2,3].

Transmission of the EHV-1 occurs through direct contact between horses; inhalation of infectious aerosols; direct contact with infected fomites, placenta and aborted foetus; or indirect contact with humans. EHV-1 first infects and replicates in the epithelial cells of the upper respiratory tract [4,5]. At this stage of EHV-1 infection, horses may present a respiratory form of disease associated with nasal discharge, cough and pyrexia. EHV-1 quickly translocates to the draining lymph nodes and associated lymphoid tissues, where it infects leucocytes [6]. Circulation of infected leucocytes during cell-associated viraemia disseminates EHV-1 through the organism to secondary sites such as the central nervous system or the reproductive tract. In these cases, EHV-1 can induce equine herpesvirus encephalomyelitis (EHM; from a mild limb ataxia to quadriplegia) or abortion, stillbirth and neonatal death [7,8,9,10]. Finally, like all herpesviruses, EHV-1 has the ability to establish latency after primo-infection and to reactivate under environmental stress conditions, immune weakness or after corticoid-based treatments [11,12,13,14,15].

EHV-1 genome is 150 kbp and carries 76 open reading frames (ORFs) encoding for proteins involved at different stages of the viral replication cycle. Among them, the 3663-kb-long open reading frame 30 (ORF30) encodes for the DNA polymerase [16]. The mechanisms conditioning one type of disease from another are not well-known, but a correlation between a non-synonymous mutation in position 2254 of ORF30 and the pathogenicity of EHV-1 strains was presented in 2006 by Nugent et al. [17]. Strains carrying a guanine (G) in position 2254 (aspartic acid (D) in position 752 of the protein), were identified as neuropathogenic, whereas those carrying an adenine (A) in the same position (asparagine (N752)) were identified as non-neuropathogenic [17,18,19]. Several studies based on field isolates have since suggested that abortion is largely associated with A_2254_ EHV-1 strains, whereas in the case of EHM, the genotype/pathotype association is less obvious [20,21,22]. It has been reported that G_2254_ EHV-1 strains replicate more efficiently in the horse and produce significantly higher viral loads [23].

Due to the diseases it causes, EHV-1 has a significant impact on equine health and the equine industry. As a preventive strategy, thorough vaccination against EHV-1 reduces the intensity of the clinical signs, virus shedding and therefore the extent of an outbreak [24]. It is generally accepted that implementation of EHV-1 vaccination in the late 1960s, alongside biosafety measures and herd management, have reduced the abortion storm occurrence [10]. To date, no vaccine exists against EHM among the various vaccines against EHV-1, possibly due to the difficulty of experimentally reproducing this form of disease [20,25,26]. Concerning the therapeutic strategy, no EHV-1 specific treatment has been commercialised. However, some studies have shown the in vitro efficacy of antiviral compounds such as ganciclovir (GCV) and aciclovir (ACV) [27,28]. The ORF30 position 2254 mutation has no effect on these molecules’ efficacy in vitro but has been shown to modify EHV-1′s sensitivity to aphidicolin (APD) in vitro. Indeed, APD had a better antiviral effect against A_2254_ (N752) EHV-1 strains than against G_2254_ (D752) EHV-1 strains [18]. However, in vivo experimental infections showed heterogeneous data after aciclovir treatment [28,29].

This study describes a new ORF30 genetic profile, with the identification of a strain carrying a cytosine (C) in position 2254, resulting in a histidine (H) in amino acid position 752. The strain was isolated at several occasions over 6 months in 2018 from one single and localised EHV-1 outbreak, during which 82 vaccinated horses were affected and showed clinical signs of respiratory diseases, associated with hyperthermia and hind limb oedema [30]. Two horses showed neurological signs of disease and one of them was euthanised. Virus isolation, complete ORF30 sequencing and multi-locus sequence typing (MLST) allowed the identification of a U_L_ clade 10 EHV-1 strain. A new PCR genotyping test was developed to identify specifically the C_2254_ strain. Experimental infection on four Welsh mountain ponies confirmed the infectious nature of the newly isolated strain. In vitro antiviral assays using real-time cell analysis (RTCA) technology [27] against the newly isolated strain and two other A/G_2254_ field strains showed that these three strains had good sensitivity to GCV, ACV and APD.

## 2. Materials and Methods

### 2.1. Sample Collection and EHV-1 Strains

One hundred and sixty samples related to EHV-1 outbreaks were collected between 2016 and 2020 from the diagnostic and equine research Institute LABÉO (Saint-Contest, France), the French Epidemiological Surveillance Network for Equine Pathology (Réseau d’Epidémio-Surveillance en Pathologie Equine (RESPE), Saint-Contest, France) and associated equine veterinary practitioners (109 nasal swabs or tracheal washes; 6 spinal cord, brain or cerebrospinal fluid samples; 36 liver, lung or placenta samples; and 9 blood samples) (Supplementary Table S1). These data included samples collected from 21 horses housed at the same premises during an EHV-1 outbreak that occurred between March and November 2018. During this outbreak, nasal swabs were taken from 9 horses (Horses 1 to 9) at the onset of clinical signs of respiratory disease. Nasal swabs, heparinised blood and serum samples were taken from 12 horses at the same time (Horses 10 to 21). Serum samples were also collected 14 days later from 11 out of these 12 horses (Horses 10 to 20).

Two other EHV-1 strains, FR-6815 (A_2254_/N_752_) and FR-38991 (G_2254_/D_752_), collected at LABÉO in 2013 and 2009 respectively, were also used in this study for antiviral assays [27].

### 2.2. Nucleic Acid Extraction

Nasal swabs were sampled in 4 mL Eagle minimal essential medium complemented with 1% antibiotics (penicillin-streptomycin-amphotericin, reference: CABPSA00-0U, Eurobio, Courtaboeuf, France) or phosphate-buffered saline (PBS). Nucleic acids from nasal swabs and from culture supernatant for the antiviral assay were extracted using QIAmp^®^ Viral RNA Mini Kit extraction kits (Qiagen, Hilden, Germany) according to manufacturer’s recommendations.

### 2.3. PCR Assays

Detection/quantitative PCR (PCR 1), a quantitative polymerase chain reaction (qPCR) assay, was performed on purified nucleic acids for EHV-1 glycoprotein B (gB) gene detection and quantification using Diallo et al. (2006) primers and probe. All qPCR details are summarised in Supplementary Table S2 [31]. TaqMan Universal PCR Master Mix (Thermo Fisher Scientific, Waltham, MA, USA) was used for this assay. A standard curve based on a cloned sequence was used for the calculation of the number of copies per sample, as described previously [27,32].

Typing PCR (PCR 2), a second real-time PCR assay, was performed using Allen et al. (2007) primers and probes for the ORF30 A/G_2254_ typing [33]. TaqMan Universal Master Mix was also used for this PCR and all details are summarised in Supplementary Table S2.

A new PCR (PCR 3) assay was designed with a specific probe for the detection of a cytosine at the ORF30 2254 position (see Results Section 3.2).

### 2.4. Sequencing Assay, MLST, Phylogenetic Analysis and 3D Modeling

ORF30 position 2254 sequencing was performed using Allen et al. (2007) PCR primers (Supplementary Table S2). Amplification PCR (PCR 4) was performed using Phusion Hot Start II DNA Polymerase (2 U/µL) chemistry (Thermo Fisher Scientific). Amplicons were purified using a QIAquick PCR Purification Kit^®^ (Qiagen). Sequencing PCR (PCR 5) was performed using a BigDye™ Terminator v3.1 Cycle Sequencing Kit (Thermo Fisher Scientific). All details are described in Supplementary Table S2. Sequencing products were purified using a ZR DNA Sequencing Clean-Up^®^ Kit (Zymo Research, Irvine, CA, USA) and electrophoresis was performed using the 3500 Genetic Analyser^®^ (Thermo Fisher Scientific). Complete ORF30 sequencing was performed by BIOFIDAL (Vaulx-en-Velin, France), as previously described [22] (Supplementary Table S3 and Figure S3). Sequences were analysed using BioEdit version 7.0.5.3 (Tom Hall, Carlsbad, CA, USA) [34]. A median joining network based on ORF30 was built using Population Analysis with Reticulate Trees (PopART) software (Jessica Leigh, David Bryant and Mike Steel, Dunedin, New Zealand) [35].

The MLST analysis was performed as previously described [22,36]. Sequences were also analysed using BioEdit, and a neighbour joining network was built using Splitstree 4 [37].

DNA polymerase three-dimensional models were built using Phyre2 and Phyre2 Investigator web servers and based on the new C_2254_ EHV-1 strain (identified as FR-56628 in Section 3.3 (accession number: MT968035)) and EHV-1 reference strains Ab4 (AY665713) and V592 (AY464052) ORF30 amino acid sequence homologies with herpes simplex virus 1 (HSV-1, also identified as human herpesvirus 1 (HHV-1)) [38].

### 2.5. Cell Culture and Virus Culture and Titration

The cell lines from RK13 (rabbit kidney cells, ATCC^®^, Molsheim, France), EEKs (equine embryonic kidney cells, kindly provided by Merial, Lyon, France) and E. Derm (equine dermal fibroblasts, ATCC^®^) were used in this study. All the cell lines were cultured at 37 °C with 5% CO_2_. RK13 cells were maintained in minimum essential medium (MEM) with Earle’s salts (reference: CM1MEM10-01, Eurobio) complemented with 1% l-glutamine (reference: CSTGLY00-0U Eurobio). EEKs were maintained using MEM Alpha (reference: L0476-500, Biowest, Nuaillé, France) complemented with 2% Lactalbumine 50× (reference 58901C, Sigma-Aldrich, St. Louis, MO, USA), 1% L-glutamine and 0.2% d-glucose 45% (reference G8769, Sigma-Aldrich). E. Derm cells were maintained in ATCC^®^ 30-2003 Eagle’s minimum essential medium (EMEM, ATCC^®^). All media were also complemented with 10% fetal bovine serum (FBS, reference: CVFSVF00-01, Eurobio) and 1% antibiotics (penicillin-streptomycin-amphotericin, Eurobio).

Peripheral blood mononucleated cells (PBMCs) were isolated from heparinised blood samples using the Hue et al. method [39] and were co-cultured with a RK13 cell monolayer for up to 6 days. Nasal swabs were sampled in 4 mL, from which 500 µL were inoculated on E. Derm and RK13 cells and cultured for up to 7 days. Co-culture was monitored using IncuCyte^®^ Live Cell Analysis technology (Essen BioScience, Ann Arbor, MI, USA). The newly isolated EHV-1 strain (FR-56628) was amplified on both E. Derm and RK13 cells. Second passage virus titer was determined on RK13, E. Derm and EEK cells lines using the Karber method [40].

EEK cells were inoculated (1.2 × 10^4^ cells per well) in three 96-well plates for xCELLigence acquisition (E-plate VIEW 96 PET, ACEA Biosciences, San Diego, CA, USA), IncuCyte^®^ acquisition and viral load quantification, respectively. Cells were cultured for 24 h prior to infection with four EHV-1 strains (the newly isolated C_2254_ EHV-1 strain, strains FR-6815 (A_2254_) and FR-38991 (G_2254_) isolated from the field, and the reference EHV-1 strain KyD (VR700^TM^, ATCC^®^)) at a multiplicity of infection (MOI) of 0.05. The time corresponding to 50% of the cell index decrease (CIT_50_) was measured [41].

### 2.6. Neutralisation Assay

A neutralisation assay was performed following the World Organisation for Animal Health (OIE) method. E. Derm cells (1.2 × 10^4^ cells per well) and the EHV-1 KyD strain (VR-700^TM^, ATCC^®^) were used for this assay [42].

### 2.7. Experimental Infection with EHV-1, Clinical Signs of Diseases, Virus Shedding and Cell-Associated Viraemia

Four 10-month-old male Welsh mountain ponies, seronegative for EHV-1 and EHV-4 (confirmed by seroneutralisaiton (SN) and complement fixing (CF) assays) and with no history of EHV infection (EHV-1 PCR was negative 3 days before infection), were experimentally infected by individual nebulisation (Flexineb^®^ nebuliser; VLC Europe, Bazoches sur Guyonne, France) [43] with the C_2254_ EHV-1 strain (third passage on RK13 cells; 2 mL dose per pony, containing a total of 5 × 10^7^ tissue culture infectious dose 50 (TCID_50_)). The day of experimental infection was defined as Day 0.

All experiments were conducted in accordance with the guidelines of the Directive 2010/63/EU of the European Parliament and of the Council, in the facilities of the EU-1277 Plateforme d’Infectiologie Expérimentale (PFIE, Infectiology of Farm, Model and Wild Animals facility, [44], INRAE, 2018, Centre Val de Loire, Nouzilly, France). All experimental procedures were approved by the Loire Valley ethical review board (CEEA VdL, committee number 19).

Rectal temperature (RT) was recorded twice a day from Day −5 to Day +21 post-infection (pi). Pyrexia was defined as a rectal temperature exceeding 38.8 °C. Clinical scores were recorded twice a day from Day −5 to Day +21 for cough, attitude, nasal discharge, ocular discharge, mandibular lymph node enlargement, ataxia and other signs of neurological disease. The cumulative clinical score after experimental infection with EHV-1 was calculated using the score for each clinical sign, according to the formula used and reported previously for equine influenza virus infection in Welsh mountain ponies, with some minor modifications [45]:

Cumulative clinical score = score RT + score nasal discharge + 2*(score cough) + score lymph node enlargement + score ocular discharge.

Nasopharyngeal swabs were collected on Day −3, from Day +1 to Day +10 and on Days +12, +14, +16, +18, +20 and +21. The swabs were placed directly into 3 mL of EMEM + 1% antibiotics. Virus DNA extraction was performed from nasopharyngeal swab extracts to measure virus shedding using the QIAamp^®^ Viral RNA Mini Kit (Qiagen) and according to the manufacturer’s instructions. Quantitative PCR was performed as described in Section 2.3. Blood samples were collected by jugular venepuncture into EDTA and dry tubes each day from Day 0 to Day +21. Virus DNA extraction from 2 mL of EDTA blood was performed to determine cell-associated viraemia, using the Nucleospin^®^ Blood L kit (Macherey-Nagel, Düren, Germany) according to the manufacturer’s instructions. Quantitative PCR (PCR 1) was performed as described in Section 2.3.

### 2.8. In Vitro Antiviral Assay

Antiviral assay was performed using xCELLigence Real-Time Cell Analysis (ACEA, Bioscience) and Incucyte^®^ technologies. Cells were infected with three EHV-1 strains (the newly isolated C_2254_ EHV-1 strain (FR-56628), and two EHV-1 strains FR-6815 (A_2254_) and FR-38991 (G_2254_) isolated from the field), as described in Section 2.5, and treated simultaneously with three antiviral drugs in a dose-response manner, as presented in Table 1. All compounds were previously dissolved at 20 mM in dimethyl sulfoxide (DMSO) and stored at −20 °C. One plate was frozen 48 h post-infection (hpi) for viral load quantification. From this plate, nucleic acids were extracted as described in Section 2.2, and viral load was quantified as described in Section 2.3. The two other plates were kept in culture for up to 140 hpi. Infected or non-infected cells treated with 0.5% DMSO were used in each plate as controls. The half maximal effective concentration (EC_50_) was calculated as previously described [27]. Experiments were carried out at least three times.

### 2.9. Statistical Analysis

For the antiviral assay results, statistical analysis was performed on StatGraphics^®^ Centurion XVI Version 16.1.12 for Windows (StatPoint Technologies, Inc., Warrenton, VA, USA) [49]. A one-way analysis of variance (ANOVA) was used to compare CIT_50_. EC_50_ were compared using Tukey’s multiple comparison test. Data were considered significant for *p* values < 0.05.

## 3. Results

### 3.1. EHV-1 Outbreak Description

In 2018, 82 horses located in a single site in France and kept in different stable blocks showed clinical signs of disease associated with EHV-1 infection (i.e., cough, nasal discharge, anorexia, pyrexia, depression and limb oedema) over a 6-month period from March 2018 to November 2018. The origin of this specific outbreak is unknown.

Due to the increased number of EHV infection cases reported to RESPE in February and March 2018 (12 outbreaks between 1 January 2018 and 31 March 2018), and rapidly after the first clinical signs appeared in a first stable block (5 March), the 64 horses kept in this stable block were monitored for a set of clinical signs of respiratory disease (e.g., cough, nasal discharge, anorexia, defined as “respiratory signs” in this report); temperature; depression and limb oedema from 5 March 2018 to the 22 April 2018. There are no data available for the first block after this date.

**Figure 1:**
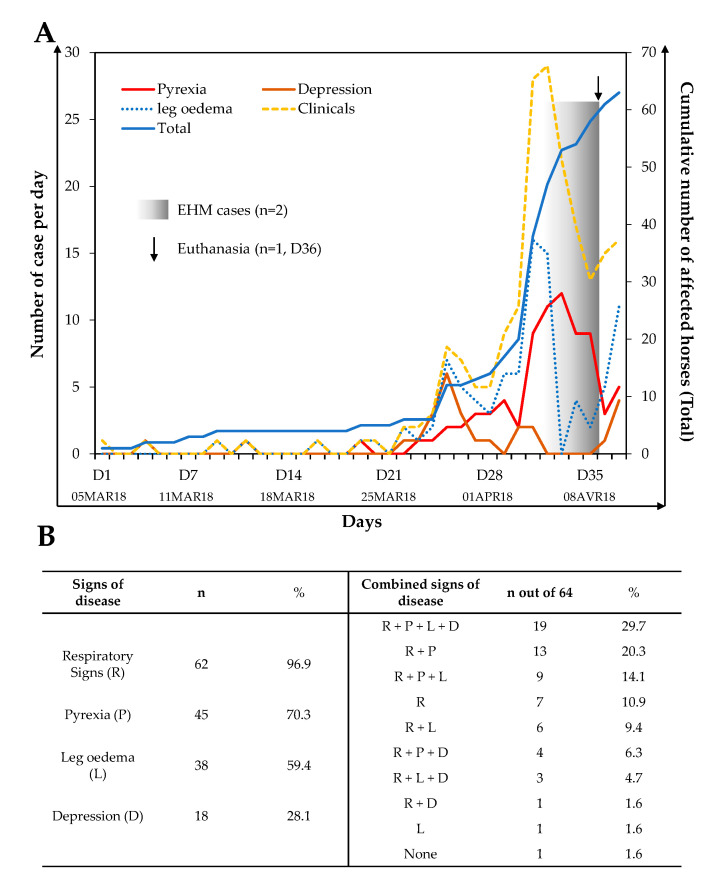
Clinical signs monitored from 5 March 2018 to 22 April 2018 in a group of 64 horses from a single stable block in the premises (**A**). The left axis represents the number of cases per day with pyrexia, depression, leg oedema or general clinical signs; the right axis represents the total number of horses affected each day (blue line). Frequency of horses showing a sign of disease, alone or in combination, is presented in (**B**).

As illustrated in Figure 1A, an increased frequency of clinical signs of disease was observed around 22 days (26 March 2018) after the first clinical signs were reported in this group. Sixty-three horses out of 64 showed clinical signs of disease during the monitoring period. The available data showed the first peak on the 25th day (29 March 2018) after the beginning of the monitoring, and this was followed by a second and major peak on the 33rd day (6 April 2018). Overall, 62 (96.9%) horses displayed respiratory signs with a duration ranging from 1 to 13 days (average: 3.2 ± 2.4 days), 45 (70.3%) horses showed pyrexia (> 38.8 °C) lasting 1 to 7 days (average: 1.8 ± 1.4 days), 38 (59.4%) horses showed limb oedema lasting 1 to 6 days (average: 2.5 ± 1.5 days) and finally 18 (28.1%) horses presented signs of depression lasting 1 to 4 days (average: 1.5 ± 1 days) (Figure 1B).

The clinical picture was variable from horse to horse, as shown in Figure 1B. Clinical signs appeared alone or in combination, with the most represented combinations being respiratory signs + pyrexia + leg oedema + depression (29.7%); respiratory signs + pyrexia (20.3%); and respiratory signs + pyrexia + leg oedema (14.1%).

Among this population, two horses showed neurological signs of disease (9 April 2018), which required euthanasia for one of them. There is no information available concerning treatments administered during the outbreak or concerning the clinical signs’ evolution after 22 April 2018.

During this period, nasal swabs were taken from four horses from the first stable block (Horses 1 to 4) and tested positive for EHV-1 using the PCR 1 assay (5 April 2018). Soon after this first episode, three other horses from a second stable block in the premises (Horses 5 to 7) were sampled and also tested positive for EHV-1 using the same PCR assay (19 April and 26 April 2018). Finally, a new episode was reported from October to November 2018, during which 14 horses from a third stable block in the premises (Horses 8 to 21) were sampled and 11 of them tested positive for EHV-1 using the PCR 1 assay (samples from Horses 12, 15 and 20 were negative; Supplementary Table S4).

### 3.2. New (ORF30) C_2254_ Mutation Identification

As described above, EHV-1 was detected in 18 nasal swab samples out of 21, all taken from different horses from the premises described previously, using a detection PCR assay (PCR 1) that amplifies a 63-bp fragment of the EHV-1 gB gene (Supplementary Table S4). As the A/G typing of the ORF30 mutation in position 2254 (PCR 2) was unsuccessful, 145 bp ORF30 fragments from the samples taken from Horses 2, 6 and 7 were sequenced, which revealed a cytosine at position 2254.

**Figure 2:**
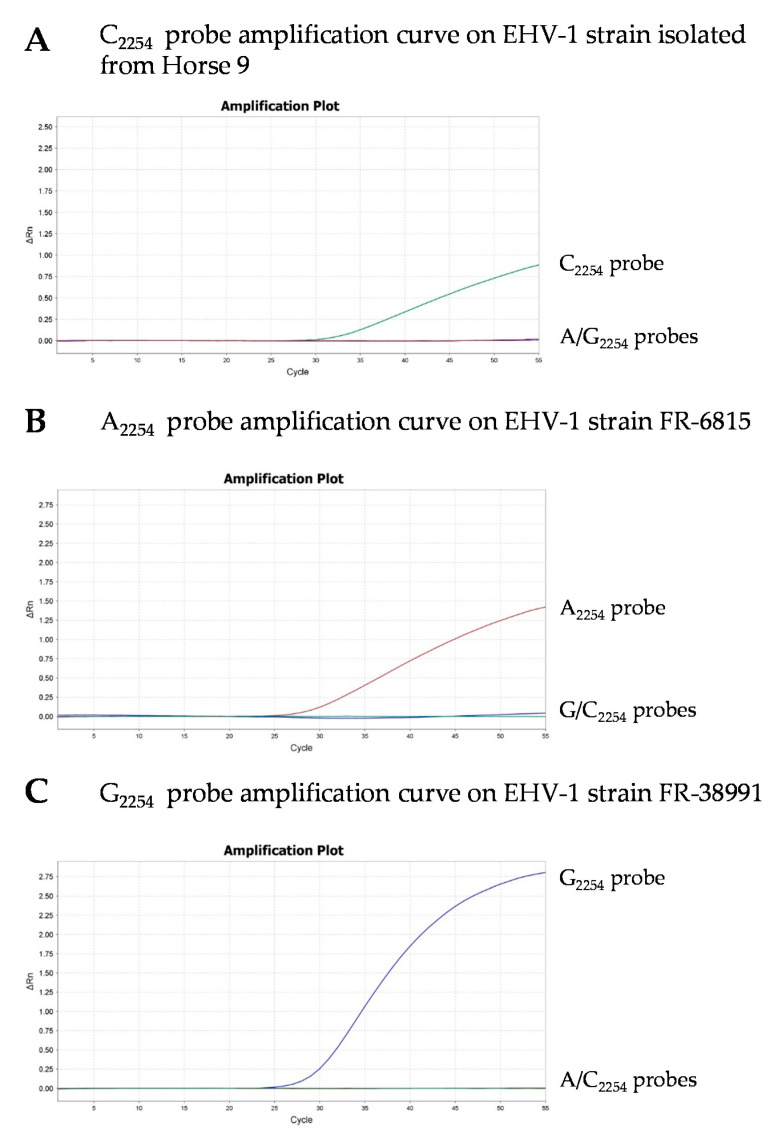
Real-time PCR amplification curves using C_2254_ probe (green curve) and both A_2254_ (red curve) and G_2254_ (blue curve) probes on a 145 bp fragment of the ORF30 for the typing of an EHV-1-positive sample from the premises described in Section 3.1 (C_2254_) (**A**), EHV-1 strains FR-6815 (A_2254_) (**B**) and FR-38991 (G_2254_) (**C**). Simplex amplification curve (C_2254_) and multiplex amplification curves (A/G_2254_) are combined in each graph.

In order to identify new C_2254_ strains using a PCR assay (PCR 3), a modified MGB probe was designed based on the existing probe sequences of Allen et al. (2007) (Table 2). Though A_2254_ and G_2254_ probes were used in a multiplex PCR (PCR 2), a C_2254_ probe was used in a simplex PCR (PCR 3), and amplification curves are shown in Figure 2. The specificity of the C_2254_ probe was validated with the EHV-1 isolates FR-6815 (A_2254_) and FR-38991 (G_2254_) and 160 DNA EHV-1 positive field samples (including the 21 samples collected in this study) obtained from different biological matrices and collected between 2016 and 2018 (Supplementary Table S1). Seventeen of the 18 EHV-1 positive (PCR 1) nasal swabs collected in 2018 from the premises described in Section 3.1 were tested and confirmed to contain C_2254_ EHV-1 strains only. These samples were the only ones to contain the C_2254_ subtype. No further samples from these premises were available (in 2019 and 2020). Thirty out of the 160 EHV-1 positive samples could not be typed due to low EHV-1 viral load. Fifty-six samples contained the A_2254_ subtype and 57 samples contained the G_2254_ subtype.

### 3.3. Virus Isolation

**Figure 3:**
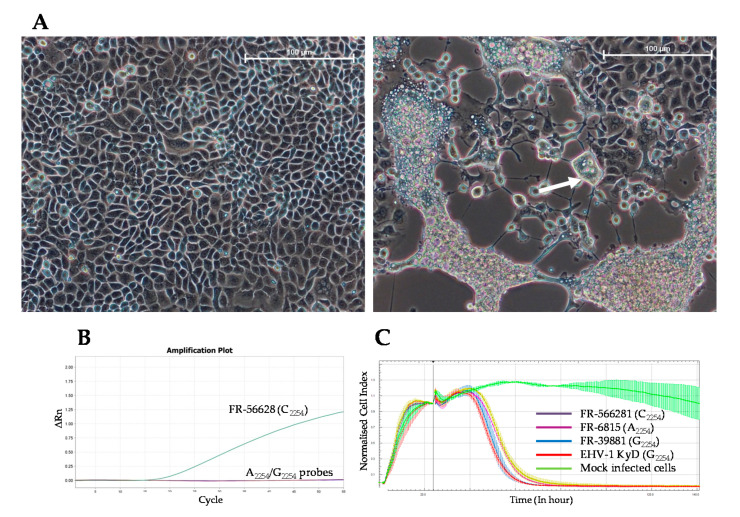
Microscopic observation of RK13 cell monolayer without (left) or with infection with the EHV-1 strain FR-56628 (120 hpi; right). Microscope lens: ×20. Scale bar: 100 µm. White arrow indicates an example of syncytia formation (**A**). Amplification curves for the EHV-1 strain FR-56628 detected with the C_2254_ probe (**B**). Real-time cell analysis of EEK cells infected with EHV-1 strains FR-56628 (C_2254_; yellow curve), FR-6815 (A_2254_; purple curve), FR38991 (G_2254_; blue curve), and reference strain KyD VR700^TM^ (G_2254_; red curve) at a multiplicity of infection (MOI) of 0.05, in comparison with mock infected cells (green curve) (**C**).

Virus isolation from three nasal swabs (Horses 2, 6 and 7) was attempted without success. Consequently, PBMCs were isolated from heparinised blood taken from six other horses (Horses 10 to 15) and co-cultured on RK13 cells. Cytopathic effects (CPEs) characterised by syncytia formation were observed for two samples (from Horses 10 and 13) after 1 and 4 days of incubation, respectively (Figure 3A). The new C_2254_ probe (PCR 3) confirmed the presence of C_2254_ EHV-1 in the culture supernatants collected after 5 days (Horse 13) and 6 days (Horse 10) of incubation (Figure 3B). The strain isolated from Horse 13 was identified as FR-56628 (MT968035). Virus isolation on an E. Derm cell monolayer from a nasal swab collected from Horse 13, at the same time, was also successful. The presence of mucosal neutralising antibodies was investigated in five nasal swabs (when sample quantity allowed it) isolated from Horses 16 to 19 and from Horse 21 in a neutralisation assay using the EHV-1 strain. No neutralisation was measured (data not shown). In vitro growth of the strain FR-56628 (C_2254_) on EEK cells was compared to three other EHV-1 strains FR-6815 (A_2254_), FR-38991 (G_2254_) and reference strain KyD VR700^TM^ (G_2254_), showing a similar decrease of the cell index for the four strains (Figure 3C). The viral load was measured and calculated in Log_10_ for each strain 48 h post-infection (hpi) on EEK cells (MOI of 0.05) (Supplementary Table S5). There was no significant variation (*p* = 0.77) of viral load 48 hpi between the EHV-1 strains FR-56628 (C_2254_/H_752_), FR-6815 (A_2254_/N_752_) and FR-38991 (G_2254_/D_752_) and KyD VR700^TM^. The viral load values in Log_10_ for each strain are as follows: 8.99 ± 0.46 (FR-56628); 9.21 ± 0.20 (FR-6815); 9.14 ± 0.18 (FR-38991); and 9.04 ± 0.07 (KyD VR700^TM^). However, there was a significant difference in the CIT_50_ mean ± standard between the EHV-1 strain FR-6815 and the EHV-1 strains FR-38991 and FR-56628 (*p* = 0.033). The CIT_50_ values for each strain are as follows: 1.11 ± 0.10 (FR-56628); 1.00 ± 0.06 (FR-6815); 1.12 ± 0.13 (FR-38991); and 1.00 ± 0.05 (KyD VR700^TM^).

### 3.4. Phylogeny

**Figure 4:**
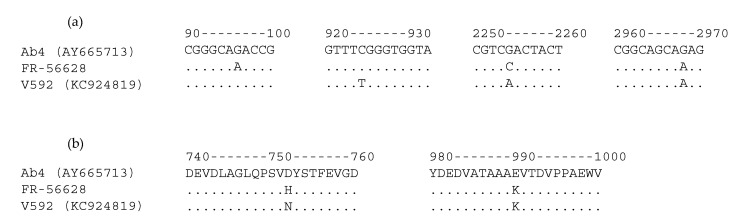
Open reading frame 30 (ORF30) nucleotide (**a**) and amino acid (**b**) sequence alignments between the EHV-1 reference strains Ab4, V592 and the newly isolated strain FR-56628.

The full ORF30 sequence was obtained from the newly isolated EHV-1 strain FR-56628 and compared with ORF30 from the EHV-1 reference strains Ab4 and V592. Three single nucleotide polymorphisms (SNPs) were identified between EHV-1 strains FR-56628 and Ab4 (positions 96, 2254 and 2968) and three SNPs were also identified between EHV-1 strains FR-56628 and V592 (positions 96, 924 and 2254), as shown in Figure 4 (complete ORF30 alignment in Supplementary Figures S5 and S6). The 2254 and 2968 SNPs are non-synonymous (D752H or N752H and E990K, respectively).

**Figure 5:**
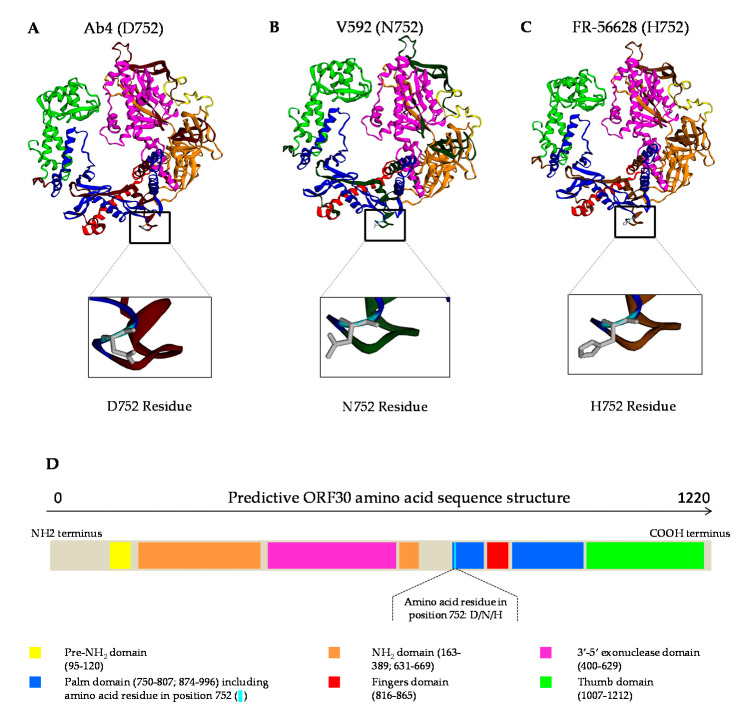
Three-dimensional models of EHV-1 strains Ab4 (D_752_) (**A**), V592 (N_752_) (**B**) and the newly isolated EHV-1 strain FR-56628 (H_752_) (**C**). ORF30 amino acid sequences based on their homology with HSV-1 DNA polymerase and predictive structure of the ORF30 amino acid sequence (**D**).

Three dimensional models of the DNA polymerase were obtained based on the homology of the ORF30 amino acid sequences from EHV-1 strains Ab4 (G_2254_), V592 (A_2254_) and FR-56628 (C_2254_) with herpes simplex virus 1 (HSV-1) (Figure 5). The amino acid residue in position 752 is located in the palm domain and remains close to the fingers domain. After analysis of the sequence by Phyre2 Investigator, the web server predicted that this residue change was unlikely to affect the protein function (data not shown).

**Figure 6:**
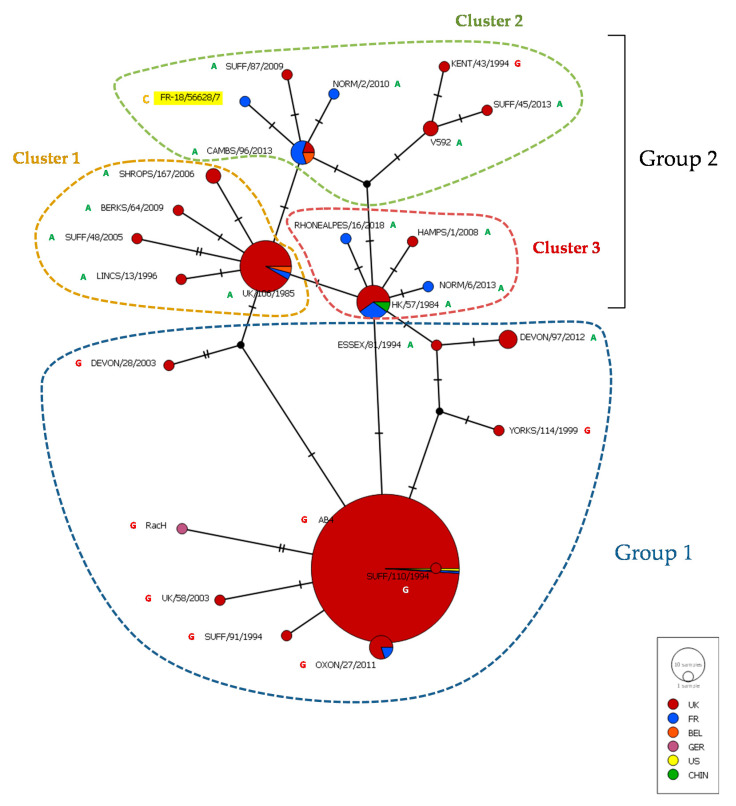
Median joining network representing ORF30 nucleotide sequences of Ab4 and V592 reference strains, 67 EHV-1 strains previously described [50] and 15 EHV-1 strains from France and Belgium [22], including the newly isolated EHV-1 strain FR-56628 (highlighted in yellow). The ORF30 position 2254 nucleotide is annotated.

The complete ORF30 gene sequence was compared with previously published sequences from 14 EHV-1 strains from France and Belgium, 67 EHV-1 strains mostly from the United Kingdom and reference strains Ab4 and V592 (Figure 6) [22,50]. As previously described, the ORF30 median joining network discriminates these strains in two groups, the second one being a group of three clusters. The EHV-1 strain FR-56628 is located in cluster 2 with one French strain isolated in 2010, three other French strains isolated between 2015 and 2018, a Belgium strain isolated in 2017 and six strains from the UK isolated between 1993 and 2013 (ORF30 maximum likelihood tree shown in Supplementary Figure S7). The EHV-1 strain FR-56628 showed one to four nucleotide differences when compared with the other EHV-1 strains from cluster 2 (including the mutation in position 2254), and from one to five nucleotide differences when compared with all 83 EHV-1 strains of the network (including the mutation in position 2254).

**Figure 7:**
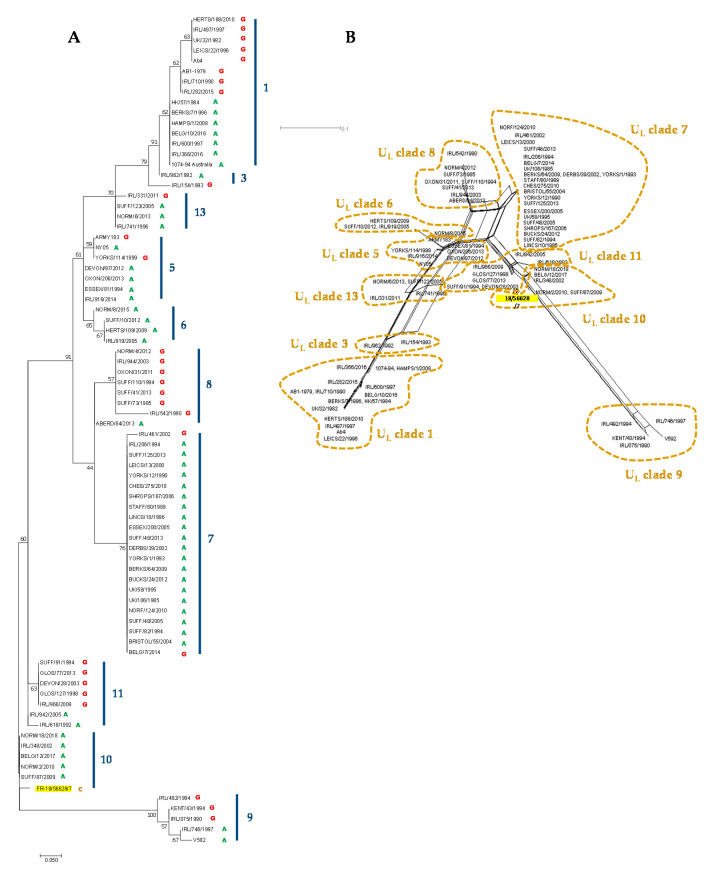
Maximum likelihood phylogenic tree based on Jones–Taylor–Thornton model (**A**) and neighbour joining network (**B**) both built from multi-locus sequence typing (MLST) amino acid sequences from 46 strains from UK, 1 strain from China, 1 strain from the US, 1 strain from Australia, 9 other strains from France and Belgium including the new strain FR-56628 (highlighted in yellow) and 22 strains from Ireland.

The EHV-1 MLST, as described by Garvey et al. (2019) [36], was also performed, and the newly isolated strain was classified as U_L_ clade 10 (Supplementary Table S8). The EHV-1 strain FR-56628 MLST amino acid sequence was compared to 79 EHV-1 strains from the UK, Ireland, China, Australia, the United States, Belgium and France and was grouped with the EHV-1 French strains NORM/2/2010, NORM18/2018, the EHV-1 Belgium strain BELG/12/2017, the EHV-1 UK strain SUFF/87/2009 and the EHV-1 Irish strain IRL/348/2002, all from U_L_ clade 10 (Figure 7). The amino acid change in ORF30 position 752 was the only feature discriminating the EHV-1 strain FR-56628 from the five other U_L_ clade 10 strains shown in this report (Supplementary Table S8). The U_L_ clade 10 EHV-1 strains NORM/2/2010, NORM/18/2018, BELG/12/2017 and SUFF/87/2009 were also grouped in ORF30 cluster 2, as shown in the ORF30 maximum likelihood tree in Supplementary Figure S7.

Two different ORF37 profiles were identified and confirmed for the EHV-1 strain FR-56628 isolated from Horse 13 (A_795_ and G_795_), which did not induce an amino acid change but which suggests that two variants of the strain were isolated (V1 and V2, Table 3). V2 was the only variant isolated from the Horse 10 sample.

### 3.5. Experimental Infection

**Figure 8:**
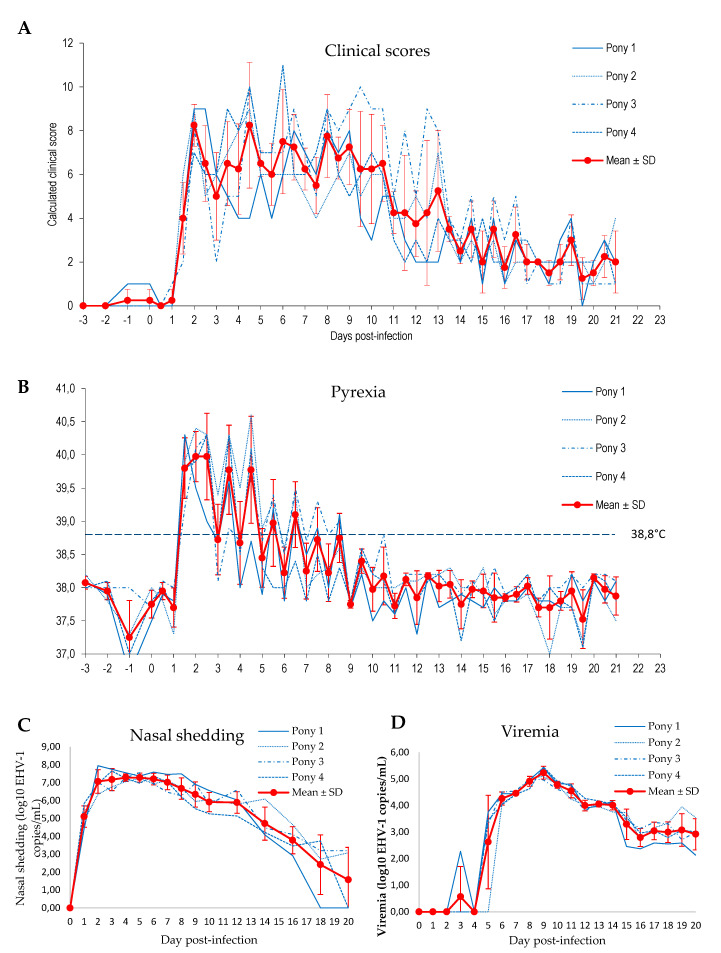
Clinical scores (**A**), pyrexia (**B**), nasal shedding (**C**) and viraemia (**D**) observed from −3 days to 21 days post-infection with EHV-1 strain FR-56628 in four Welsh ponies.

Four 10 month-old Welsh ponies were experimentally infected by nebulisation with EHV-1 strain FR-56628 (C_2254_). Daily clinical scores were evaluated twice a day. As illustrated in Figure 8A and Supplementary Figure S9, all ponies showed a significant increase (*p* < 0.0001, multifactiorial ANOVA with days as main effect and pony ID as covariates) of the cumulative clinical score from day 1 post-experimental infection, from the second checking point of the day. Overall, the cumulative clinical score started to decrease 10 to 14 days post-infection, depending on the individual, but remained above pre-infection level until the end of the study (i.e., 21 days after experimental infection). Pyrexia (i.e., body temperature >38.8 °C) was measured in all ponies on day 1.5 post-infection, on the second checking point of the day (Figure 8B). Abnormal body temperatures were mostly recorded in the afternoon and lasted from 4 to 8 days, with peaks of temperature up to 40.6 °C. Nasal discharge was first observed on day 1 and was recorded until the end of the study. Cough was recorded in all ponies on day 2 post-infection and intermittently thereafter. Mandibular lymph node enlargement was observed for one pony on day 2, and all four ponies from day 3 to day 11. All ponies showed signs of lethargy on day 2, and an overall reduction in food consumption was also noted during the first week post-infection. Finally, tail hypotonia was observed for all ponies, from day 2 post-infection and lasted from 4 to 5 days.

Viral load quantification showed that virus shedding started on day 1, with a peak between days 2 and 6, depending on the pony (Figure 8C). From day 7, nasal shedding started to decrease but EHV-1 remained detectable in nasopharyngeal swabs from all ponies on day 16 post-infection, and was still detected in two out of four ponies on day 20. EHV-1 was detected in the blood sample collected from one pony on day 3, from three out of four ponies on day 5 and in blood samples from all four ponies from day 6 to the end of sampling (day 20). The peak was measured on day 9 (Figure 8D).

### 3.6. Antiviral Assay

The activity of three antiviral compounds (aciclovir, aphidicolin and ganciclovir) was measured against three EHV-1 strains carrying the C_2254_ (H_752_), A_2254_ (N_752_) and G_2254_ (D_752_) mutations (FR-56628, FR-6815 and FR-38991, respectively) and results are shown in Table 4. RTCA curves and viral load at 48 hpi for each concentration of ganciclovir, aciclovir and aphidicolin against EHV-1 strain FR-56628 (C_2254_) are shown in Supplementary Figure S10. Concerning the antiviral assay, results obtained by qPCR and RTCA showed that aphidicolin was the most effective compound against the three EHV-1 strains, whereas aciclovir was the least effective. There was no significant difference of sensitivity (*p* > 0.05) between the three EHV-1 strains (FR-56628 (C_2254_/H_752_), FR-6815 (A_2254_/N_752_) and FR-38991(G_2254_/D_752_)) to ganciclovir and aphidicolin according to EC_50_ values obtained from the qPCR assay. When measured by impedancemetry, the EHV-1 strains FR-38991 (G_2254_/D_752_) appeared to be more sensitive (*p* = 0.046) to aphidicolin than the FR-6815 (A_2254_/N_752_) strain. According to EC_50_ obtained from the qPCR assay, the FR-56628 (C_2254_/H_752_) and FR-6815 (A_2254_/N_752_) strains were significantly more sensitive to aciclovir (*p* = 0.012 and 0.036, respectively) than FR-38991 (G_2254_/D_752_). There was no significant difference in sensitivity between FR-56628 and FR-6815 compared with aciclovir using the qPCR assay. Impedance measurements showed that FR-56628 was significantly more sensitive to aciclovir (*p* = 0.019) than FR-6815, but showed no significant difference of sensitivity between FR-56628 and FR-38991, and between FR-6815 and FR-38991.

## 4. Discussion

Equid herpesvirus 1, which was first designated as equine abortion virus [51] is a major cause of infectious abortion and can also cause sporadic EHM cases, in addition to usually mild respiratory infection. In 2006, Nugent et al. suggested that ORF30 position 2254 could discriminate a neuropathogenic genotype (G_2254_/D_752_) from a non-neuropathogenic genotype (A_2254_/N_752_), although the mechanisms involved are not well defined [17,20]. The current study reports the discovery of a third genotype with the isolation of a C_2254_/H_752_ strain in one single site in France. The EHV-1 strain affecting these premises was isolated in 2018 and a new PCR assay was designed for the detection of the C_2254_ mutation and successfully discriminated A/G and C_2254_ strains, in combination with a PCR assay designed by Allen et al. [33]. The C_2254_ mutation was not detected in strains from other premises in France between 2016 and 2020. Due to the absence of breeding activity on the affected premises, and the absence of further C_2254_/H_752_ EHV-1 outbreaks since 2018, it is not possible to evaluate the effect of the C_2254_/H_752_ EHV-1 strain in pregnant mares at the current time. In addition, the site from which the strain was isolated is very restrictive on horse mixing and circulation, which could explain the absence of identification of any other C_2254_ strains since the 2018 outbreak. It should also be taken into account that 2018 was a year with a large number of EHV outbreaks and consequently with a large number of samples taken. The number of EHV outbreaks in France and associated sampling is usually much lower, limiting opportunities for virus isolation. Despite anecdotal reports of frequent vaccination in these premises (every 6 months), there was no information available on the immune status of the horses at the time of infection. Vaccine efficacy against this strain would need to be further investigated. Isolation from nasal swabs was not as successful as from co-culture with PBMCs, which could be explained by the possible presence of interferon alpha (INF-α) in infected horses’ nasal secretions, as shown in 2010 by Gryspeerdt et al. [52]. When cultured in vitro, the strain induced cytopathic effects (CPEs) associated with polykaryocytes and syncytia formation, as usually observed by microscopy in cell culture after infection with EHV-1 [53]. The new EHV-1 strain, named FR-5628, questions the effect of ORF30 position 2254 mutation on the strain activity.

**Figure 9:**
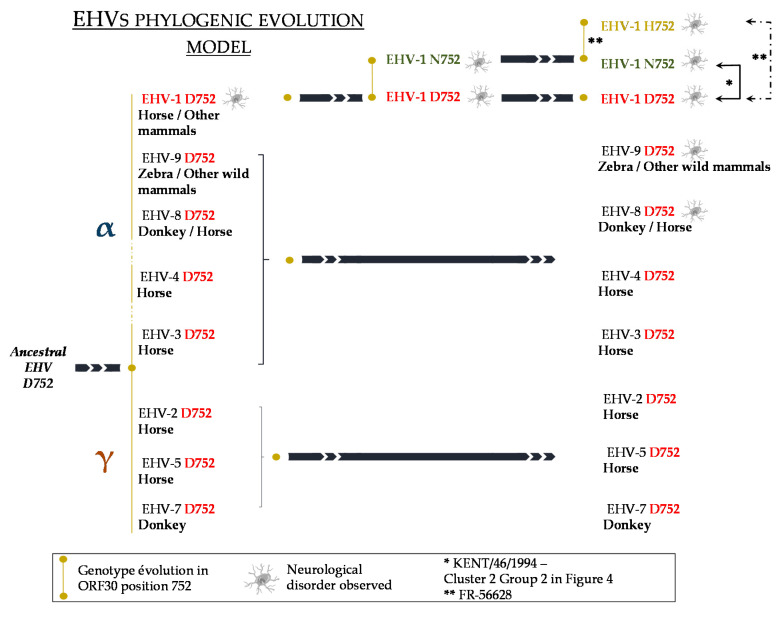
Possible evolutionary model for equid herpesviruses. Reverse evolution is shown with black arrows and illustrated with strains from the current study (KENT/46/1994 (*) and FR-56628 (**)).

Based on the amino acid in position 752 (or equivalent) of DNA polymerase gene comparison between several herpesviruses, including the eight known equid herpesviruses (EHV-1, 2, 3, 4, 5, 7, 8 and 9), as already pointed out by Nugent et al. (2006), D_752_ (or an equivalent position in other herpesviruses) appears to be a highly conserved amino acid amongst the Herpesviridae family (Supplementary Figure S11) [17]. It has been suggested that D_752_ strains are more likely to be ancestral strains from which N_752_ would have evolved, as shown in Figure 9, in which the G_2254_ strain KENT/64/1994 (see Figure 6) was proposed to have evolved from an A_2254_ strain from group 2 cluster 2. All of the sequences compared carried an aspartic acid (D) at this position, with the exception of numerous EHV-1 strains (with V592 as a representative N_752_ EHV-1 strain) and EHV-1 FR-56628, the only H_752_ strain described to date [30]. To our knowledge, asparagine (N) and histidine (H) are the only two other amino acids identified in equid herpesviruses’ DNA polymerase gene in position 752. In addition, a study showed that N_752_ strains (isolated from abortion) could infect equally monocytic CD172a+ cells and T lymphocytes, whereas D_752_ strains (isolated from neurological cases) could infect CD172a+ cells in a larger proportion than T lymphocytes. In accordance with other studies, the authors suggested that N_752_ had adapted its immune evasion strategy after co-evolving from D_752_ strains with the host immune system [52,54,55]. Concerning the genotype/pathotype association suggested by Nugent et al. (2006), it was followed by a statistical report by Perkins et al. performed on 176 EHV-1 isolates, showing a strong but not strict association between G_2254_/D_752_ strains and neurological disease, and A_2254_ strains and abortion disease. According to the authors, the probability of neurological disease being associated with G_2254_/D_752_ strains was 162 times greater than the probability of it being associated with A_2254_/N_752_ strains [56]. Several studies then showed that this genotype/pathotype association is not exclusive [21,22,57,58,59]. The discovery of a third genotype C_2254_/H_752_ and its potential association with neurological signs makes the understanding of the genotype/pathotype association even more complex. EHV-1 is the only equid herpesvirus known to have possibly evolved from D_752_ strains to N_752_ and H_752_ strains. Along with EHV-8 and EHV-9, these three viruses are also the only equid herpesviruses to our knowledge that have been reported as causing neurological diseases. This observation is in line with other statements suggesting that the amino acid in position 2254 is not the only determinant for neurovirulence and could also depend on the environment (air quality, stress, training management, etc.), the immune status of the horse and its genetic background [9,56,60].

An antiviral assay with the newly isolated FR-56628 EHV-1 strain and two other A/G_2254_ strains was performed with ganciclovir, aciclovir and aphidicolin. Ganciclovir and aciclovir are two nucleoside analogues (analogues to deoxyguanosine). Indeed, triphosphorylated ganciclovir and aciclovir act as competitive nucleotide substrates for DNA polymerase [46,47]. When phosphorylated, first by the viral thymidine kinase (TK) and then by the cellular TK, ganciclovir and aciclovir are incorporated in the synthesised DNA strand. Replication stops either immediately after treatment (aciclovir), or after the incorporation of several nucleotides (ganciclovir) [61]. Aphidicolin is a tetracyclic diterpen that has been described to inhibit the activity of B-family DNA polymerase (to which EHV-1 DNA polymerase belongs) as a competitive dNTP binding site molecule [62]. Some studies have shown a good efficacy of this molecule against human herpesviruses, especially in strains resistant to nucleoside analogues [63,64,65]. The herpesvirus DNA polymerase crystal structure has only been described for HSV-1. However, as explained in 2007 by Goodman et al., the EHV-1 amino acid D_752_ (G_2254_) residue is homologous to HSV-1 amino acid D751 residue, which is located in the palm domain, a region close to the dNTP binging domain (fingers domain) [18,66,67]. In addition, in their in vivo study, Goodman et al. (2007) suggest that amino acid change from D_752_ (G_2254_) to N_752_ (A_2254_) could alter DNA polymerase activity, after they highlighted thst aphidicolin was less effective against DNA polymerase N_752_ (A_2254_) mutant and N_752_ (A_2254_) strains than against DNA polymerase D_752_ (G_2254_) mutant and D_752_ (G_2254_) strains [18]. After binding with dNTP, the DNA polymerase subdomain rotates and changes conformation to allow dNTP incorporation [66,68]. Aphidicolin may change the fingers and palm domain interaction, as it is associated with Human Pol α by Baranosvki et al. [62]. The effect of an amino acid change in the palm domain on the interaction of aphidicolin (but also possibly of ganciclovir and aciclovir) with the DNA polymerase dNTP binding site was therefore questioned. In this study, aphidicolin and ganciclovir showed a good in vitro effect against the three EHV-1 strains (FR-56628, FR-6815 and FR-38991), whereas a higher concentration of aciclovir was required to inhibit these three strains’ replication. As both ganciclovir and aciclovir require the viral TK for their first phosphorylation, it is unlikely that aciclovir’s lower efficacy was due to strain TK resistances. The 3D models of the EHV-1 DNA polymerase, based on the HSV-1 DNA polymerase model, did not predict an effect of an amino acid change in position 752 on the protein function. In addition, antiviral assays did not show an evident influence of position 752 amino acid residue on the efficacy of either ganciclovir or aphidicolin in vitro (no significant EC_50_ difference was found between FR-56628 and FR-6815, and between FR-56628 and FR-38991). EC_50_, measured by impedance measurements, showed that aphidicolin was significantly more efficient against FR-38991 (G_2254_/D_752_) than against FR-6815 (A_2254_/N_752_), but this was not confirmed by the qPCR assay. Even if a good correlation between RTCA and qPCR EC_50_ values has been demonstrated, the impedance method depends on parameters that are more variable than the qPCR assay, which can explain the differences [69]. Aciclovir showed differences in efficacy against the three strains which were not reproduced with the two methods. As nucleotide position 2254 is not the only site where non-synonymous mutations occur in ORF30, these could also imply changes of DNA polymerase conformation, different from the strains used by Goodman et al. (2007) and constitute a possible explanation for the aphidicolin effects observed in the two studies.

When compared to 83 other EHV-1 strains, the complete ORF30 nucleotide sequence of the newly isolated EHV-1 strain FR-56628 showed that, in addition to the mutation in position 2254, this part of the genome had from zero to three other mutations, when compared to the other EHV-1 strains in group 2 cluster 2. This cluster is mainly represented by A_2254_ strains, with the exception of one G_2254_ strain and the FR-56628 C_2254_ strain. As all other G_2254_ strains are clustered in the ORF30 group 1, the possibility is not excluded that some EHV-1 A_2254_ strains from this cluster (group 2) reversed to EHV-1 G_2254_ strains or evolved to a C_2254_ strain (Figure 9). Considering the amino acid sequence, the EHV-1 MLST placed the EHV-1 strain FR-56628 among U_L_ clade 10, alongside five EHV-1 strains also classified into ORF30 group 2 cluster 2. The amino acid change in ORF30 position 752 differentiates the strain FR-56628 MLST sequence from the other members of the U_L_ clade 10 used in this study. However, the nucleotide MLST sequence not only showed another silent variation site in ORF37, but also suggested that two different FR-56628 variants have been isolated from Horse 13. ORF37 is a conserved gene among alphaherpesviruses and encodes the HSV-1 UL24 homolog [16]. On the contrary to ORF30, this gene has been classified among the non-essential genes for growth on cultured cells [70,71]. HSV-1 UL24 has been shown to play a role in pathogenicity and to affect in vivo and in vitro replication and reactivation from latency [72,73]. This protein seems to carry numerous functions, such as viral gene expression regulation, dispersion of cellular proteins in the nucleus and glycoproteins distribution in the cytoplasm [74,75]. It was also shown to be required for EHV-1 pathogenicity in a mouse model [70]. Numerous transcription initiation sites and therefore transcripts have been identified for HSV-1 UL24. The impact of the silent mutation detected in this study on different transcripts from different transcription initiation sites is unknown, although all the ATG codons from EHV-1 ORF37 seemed to be in the same reading frame. This silent mutation was not identified in the EHV-1 strain isolated from Horse 10, but no information is available concerning other horses, making it difficult to date the mutation event. Indeed, the strain could either have mutated after infecting Horse 13, or this could also be the result of a double infection with the two variants, after physical contact with different horses.

Experimental infection of four 10-month-old Welsh ponies with EHV-1 strain FR-56628 (C_2254_) induced characteristic clinical signs of EHV-1 infection in all ponies starting from Day 1 post-infection. None of the horses showed neurological clinical signs, but EHM is difficult to reproduce in experimental infections [7,76,77,78]. However, the rapid onset of clinical signs, nasal shedding and viraemia were in accordance with previous experimental challenges performed using EHV-1 strains V592 (A_2254_) and Ab4 (G_2254_) with slightly lower (10^5.5^ TCID50 and 10^6.6^ TCID50, for example) or similar (10^7.5^, 10^7.6^, for example) infectious doses [6,7,77,79].

## 5. Conclusions

This study describes the isolation of a new 2254 mutant strain (C_2254_/H_752_) from a unique site in France in 2018 which was associated with a large number of affected animals, despite frequent vaccination. There was no other identification or isolation of C_2254_ EHV-1 strains before 2018, nor in 2019 and 2020. The pathogenicity of this strain was confirmed in the context of an in vivo experimental infection that highlighted the rapid onset of disease and significant excretion and cell-associated viraemia. The impact of the mutation on ganciclovir, aciclovir and aphidicolin susceptibility was limited. The molecular characterisation of the strain, the predictive 3D model of the ORF30 and the in vitro culture taken altogether did not show evidence of a major effect of this new C_2254_ mutation on the strain’s behaviour. Most equine herpesviruses have the D_752_ mutation. EHV-1 is the only equine herpesvirus to carry the N_752_ or H_752_ mutations. This is why ORF30 and MLST phylogeny tend to indicate that this C_2254_/H_752_ has evolved from N_752_ strains.

## Figures and Tables

**Table 1:** Antiviral molecules tested against the newly isolated equid herpesvirus 1 (EHV-1) strain (FR-56628) and EHV-1 strains FR-6815 and FR-38991.

Antiviral Molecule Name	Abbreviation	Empirical Formula	DNA Polymerase Inhibitor	Initial Concentration	Final Concentration	Dilution Fold	Replicates
Ganciclovir	GCV	C_9_H_13_N_5_O_4_	Nucleoside analogue ^1^	50 µM	0.10 µM	2	3
Aciclovir	ACV	C_8_H_11_N_5_O_3_	Nucleoside analogue ^2^	100 µM	0.78 µM	2	3
Aphidicolin	ADP	C_20_H_34_O_4_	Nucleoside-binding site homology ^3^	2.5 µM	0.02 µM	2	4

^1^ Elion et al. 1983 [46], ^2^ Matthews and Boehme 1988 [47], ^3^ Sheaff et al. 1991 [48].

**Table 2:** PCR conditions of the newly developed assay (PCR 3) for C_2254_ strain detection.

Primers and Probe	Sequence
Primer GaF	5′-CCACCCTGGCGCTCG-3′
Primer GaR	5′-AGCCAGTCGCGCAGCAAGATG-3′
Probe C_2254_	5′- NED-CATCCGTCCACTACTC-MGB-NFQ-3′
Thermoprofile	95 °C during 10 min, 55 cycles x {95 °C during 15 s, 65 °C during 1 min} — QuantStudio™ 12K Flex Real-time PCR System
Mixture for 1 sample	2.5 µL DNA extract + 12.5 μL TaqMan Universal PCR Master Mix + 0.6 μL of each primer (20µM) + 0.5 µL of 10µM probe (depending on titration) + nuclease free water to complete to 25 µL

**Table 3:** MLST codon alignment between reference strains Ab4 and V592, and strain FR-56628 variants V1 and V2, based on MLST. FR-56628 nucleotides in ORF30 position 2254 and ORF37 are highlighted in grey.

Strain	U_L_ Clade	ORF	2	5	8	11	11	13	13	13	13	13	13	14		14	14	15	22	29	30	30	31	32	33	33	34	36	37	39	40	42	45	46	50	52	57	73	76
		nt	175	340	340	565	703	913	1213	1378	1474	1477	1495	1852–1860		1882	2074	496	1288	34	2254	2968	268	124	43	2926	196	139	793	1318	586	3823	1279	418	1009	1156	2410	364	382
Ab4	1		GGC	GGC	GAC	CAG	AGG	TCA	GCA	GCC	GAA	ACT	GCA	---		AGA	AGC	GAT	TCC	ACG	GAC	GAG	AAC	TCG	AAT	AAT	GAT	AGC	GCG	TCA	CGT	AAG	GAA	TTT	CCG	GCC	AAG	GCC	TTT
FR-56628 V1	10		.A.	.T.	...	...	...	.T.	...	A..	...	...	...	CCCCAGCCG		...	...	A..	...	...	C..	A..	.G.	...	C..	G..	...	...	.TA	...	...	...	.G.	.C.	...	.T.	.G.	...	.C.
FR-56628 V2	10		.A.	.T.	...	...	...	.T.	...	A..	...	...	...	CCCCAGCCG		...	...	A..	...	...	C..	A..	.G.	...	C..	G..	...	...	.T.	...	...	...	.G.	.C.	...	.T.	.G.	...	.C.
V592	9		.A.	.T.	A..	A..	...	.T.	...	A..	...	...	...	CCCCAGCCG		...	...	A..	C..	.A.	A..	A..	.G.	.T.	C..	G..	.G.	C..	.T.	.T.	.A.	.G.	.G.	.C.	T..	.T.	.G.	.T.	.C.

nt = nucleotide position.

**Table 4:** EC_50_ (µM) ± standard deviation (SD) of ganciclovir (GCV), aciclovir (ACV) and aphidicolin (APD) against three different EHV-1 ORF30 2254 variants measured by qPCR assays and real time cell analysis (RTCA).

Strains	GCV		ACV		APD	
	qPCR	RTCA	qPCR	RTCA	qPCR	RTCA
FR-56628 (C_2254_/H_752_)	0.57 ± 0.2	1.29 ± 0.69	7.87 ± 0.98 ^a^	30.32 ± 3.89 ^c^	0.05 ± 0.03	0.14 ± 0.07
FR-6815 (A_2254_/N_752_)	0.75 ± 0.44	2.49 ± 1.20	14.15 ± 5.27 ^b^	56.19 ± 11.04 ^c^	0.09 ± 0.04	0.19 ± 0.03 ^b^
FR-38991 (G_2254_/D_752_)	0.73 ± 0.42	1.20 ± 0.55	35.98 ± 10.97 ^a,b^	35.09 ± 10.32	0.09 ± 0.04	0.09 ± 0.05 ^b^

^a^ Significant difference between strain FR-56628 (C_2254_/H_752_) and strain FR-38991 (G_2254_/D_752_) (*p* < 0.05). ^b^ Significant difference between strain FR-6815 (A2_254_/N_752_) and strain FR-38991 (G_2254_/D_752_) (*p* < 0.05). ^c^ Significant difference between strain FR-56628 (C_2254_/H_752_) and strain FR-6815 (A_2254_/N_752_) (*p* < 0.05).

## Supplementary Materials

The following are available online at https://www.mdpi.com/1999-4915/12/10/1160/s1, Table S1: ORF30 position 2254 typing using the three different probes (A2254, G2254 and C2254) in order to check the probe C2254 specificity. Table S2: PCR 1, 2, 4 and 5 primers and probe sequences, thermoprofiles and mixtures. Table and Figure S3: complete ORF30 sequencing primers (A) and their distribution on ORF30 (B). Table S4: PCR results, viral load and ORF30 position 2254 subtype in the 14 nasal swabs collected on the premises described in Section 3.1. Figure S5: ORF30 nucleotide alignment of the EHV-1 strains Ab4 (AY665713), FR-56628 (MT968035) and V592 (AY464052). Figure S6: ORF30 amino acid alignment of the EHV-1 strains Ab4 (AY665713), FR-56628 (MT968035) and V592 (AY464052). Figure S7: Maximum likelihood tree based on Tamura–Nei model and built using ORF30 sequences of Ab4 and V592 reference strains, 67 EHV-1 strains previously described (Bryant et al. 2018), 14 EHV-1 strains from France and Belgium (Sutton et al. 2019) and the EHV-1 strain FR-56628 (highlighted in yellow). Table S8: EHV-1 strain FR-56628 MLST amino acid sequence compared to EHV-1 reference strains Ab4 and V592 (A) and to UL clade 10 EHV-1 strains (B). Figure S9: Number of horses experimenting nasal discharge/cough/ocular discharge (A), mandibular lymph node enlargement (B) and pyrexia/attitude (C). Figure S10: Antiviral analysis of the effect of (A) GCV, (B) ACV and (C) APD using (i) impedance measurement, (ii) viral load quantification. Figure S11: Amino acid in ORF30 position 752 (or equivalent region) of 18 herpesviruses (A). Conserved amino acids on a 106-bp region of 18 herpesviruses DNA polymerase genes are highlighted in shades of blue (when 9/18 or more herpesviruses contained the same amino acid). Aspartic acids in position 752 or equivalent are highlighted in red and variants are highlighted in yellow (B).
