# Supplementary material for: Identification of a New Equid Herpesvirus 1 DNA Polymerase (ORF30) Genotype with the Isolation of a C2254/H752 Strain in French Horses Showing no Major Impact on the Strain Behaviour
Source: Viruses. 2020 Oct 13;12(10):1160. doi: 10.3390/v12101160 (PMC7650556; doi:10.3390/v12101160)
Supplement: Supplementary file 1 [file viruses-12-01160-s001.zip › Sutton et al 2020 EHV-1 Viruses Supplementary Material-1 11-10-2020.pptx]

## Slide 1
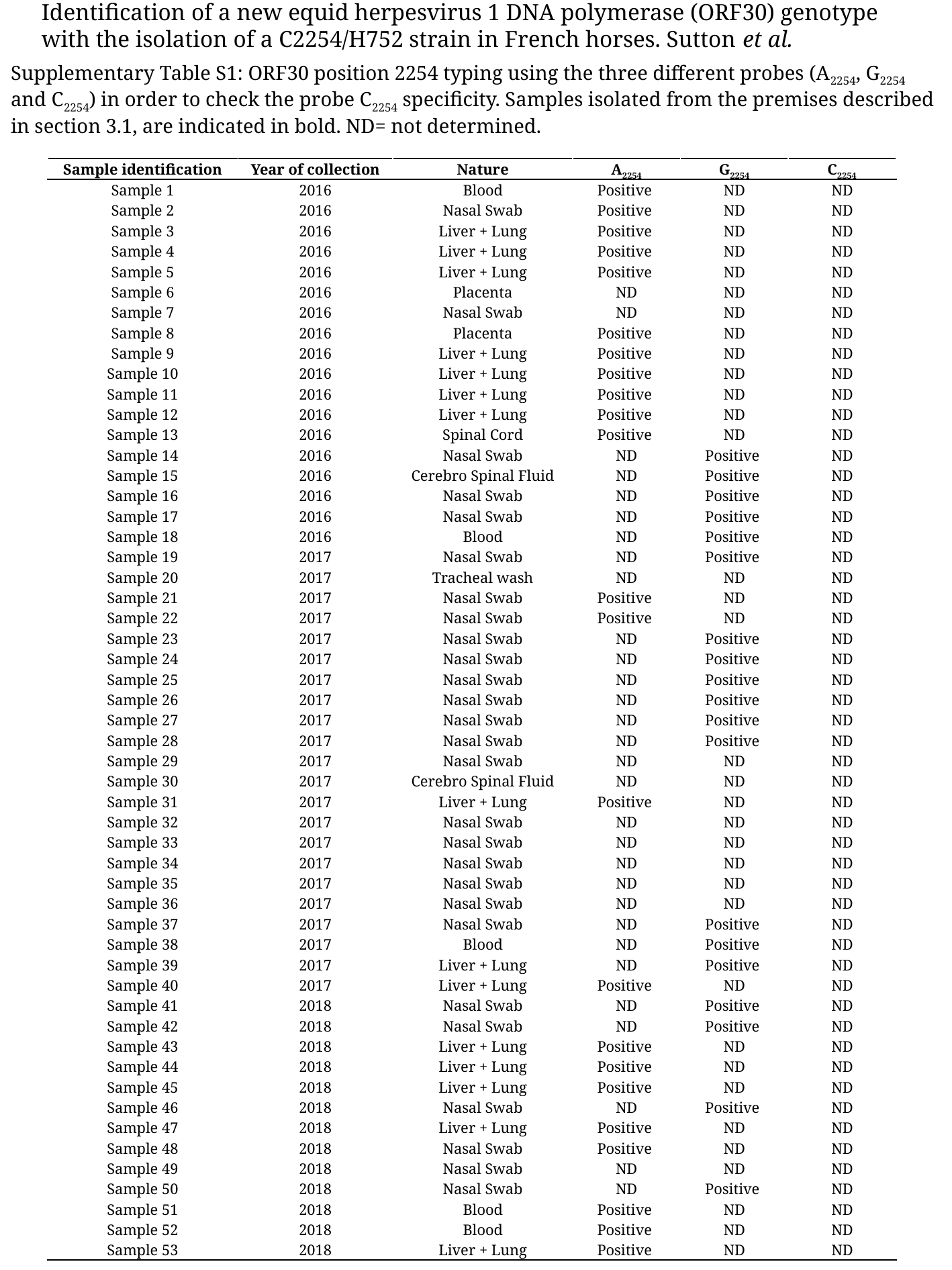

Identification of a new equid herpesvirus 1 DNA polymerase (ORF30) genotype with the isolation of a C2254/H752 strain in French horses. Sutton et al.
Supplementary Table S1: ORF30 position 2254 typing using the three different probes (A2254, G2254 and C2254) in order to check the probe C2254 specificity. Samples isolated from the premises described in section 3.1, are indicated in bold. ND= not determined.
| Sample identification | Year of collection | Nature | A2254 | G2254 | C2254 |
| --- | --- | --- | --- | --- | --- |
| Sample 1 | 2016 | Blood | Positive | ND | ND |
| Sample 2 | 2016 | Nasal Swab | Positive | ND | ND |
| Sample 3 | 2016 | Liver + Lung | Positive | ND | ND |
| Sample 4 | 2016 | Liver + Lung | Positive | ND | ND |
| Sample 5 | 2016 | Liver + Lung | Positive | ND | ND |
| Sample 6 | 2016 | Placenta | ND | ND | ND |
| Sample 7 | 2016 | Nasal Swab | ND | ND | ND |
| Sample 8 | 2016 | Placenta | Positive | ND | ND |
| Sample 9 | 2016 | Liver + Lung | Positive | ND | ND |
| Sample 10 | 2016 | Liver + Lung | Positive | ND | ND |
| Sample 11 | 2016 | Liver + Lung | Positive | ND | ND |
| Sample 12 | 2016 | Liver + Lung | Positive | ND | ND |
| Sample 13 | 2016 | Spinal Cord | Positive | ND | ND |
| Sample 14 | 2016 | Nasal Swab | ND | Positive | ND |
| Sample 15 | 2016 | Cerebro Spinal Fluid | ND | Positive | ND |
| Sample 16 | 2016 | Nasal Swab | ND | Positive | ND |
| Sample 17 | 2016 | Nasal Swab | ND | Positive | ND |
| Sample 18 | 2016 | Blood | ND | Positive | ND |
| Sample 19 | 2017 | Nasal Swab | ND | Positive | ND |
| Sample 20 | 2017 | Tracheal wash | ND | ND | ND |
| Sample 21 | 2017 | Nasal Swab | Positive | ND | ND |
| Sample 22 | 2017 | Nasal Swab | Positive | ND | ND |
| Sample 23 | 2017 | Nasal Swab | ND | Positive | ND |
| Sample 24 | 2017 | Nasal Swab | ND | Positive | ND |
| Sample 25 | 2017 | Nasal Swab | ND | Positive | ND |
| Sample 26 | 2017 | Nasal Swab | ND | Positive | ND |
| Sample 27 | 2017 | Nasal Swab | ND | Positive | ND |
| Sample 28 | 2017 | Nasal Swab | ND | Positive | ND |
| Sample 29 | 2017 | Nasal Swab | ND | ND | ND |
| Sample 30 | 2017 | Cerebro Spinal Fluid | ND | ND | ND |
| Sample 31 | 2017 | Liver + Lung | Positive | ND | ND |
| Sample 32 | 2017 | Nasal Swab | ND | ND | ND |
| Sample 33 | 2017 | Nasal Swab | ND | ND | ND |
| Sample 34 | 2017 | Nasal Swab | ND | ND | ND |
| Sample 35 | 2017 | Nasal Swab | ND | ND | ND |
| Sample 36 | 2017 | Nasal Swab | ND | ND | ND |
| Sample 37 | 2017 | Nasal Swab | ND | Positive | ND |
| Sample 38 | 2017 | Blood | ND | Positive | ND |
| Sample 39 | 2017 | Liver + Lung | ND | Positive | ND |
| Sample 40 | 2017 | Liver + Lung | Positive | ND | ND |
| Sample 41 | 2018 | Nasal Swab | ND | Positive | ND |
| Sample 42 | 2018 | Nasal Swab | ND | Positive | ND |
| Sample 43 | 2018 | Liver + Lung | Positive | ND | ND |
| Sample 44 | 2018 | Liver + Lung | Positive | ND | ND |
| Sample 45 | 2018 | Liver + Lung | Positive | ND | ND |
| Sample 46 | 2018 | Nasal Swab | ND | Positive | ND |
| Sample 47 | 2018 | Liver + Lung | Positive | ND | ND |
| Sample 48 | 2018 | Nasal Swab | Positive | ND | ND |
| Sample 49 | 2018 | Nasal Swab | ND | ND | ND |
| Sample 50 | 2018 | Nasal Swab | ND | Positive | ND |
| Sample 51 | 2018 | Blood | Positive | ND | ND |
| Sample 52 | 2018 | Blood | Positive | ND | ND |
| Sample 53 | 2018 | Liver + Lung | Positive | ND | ND |

## Slide 2
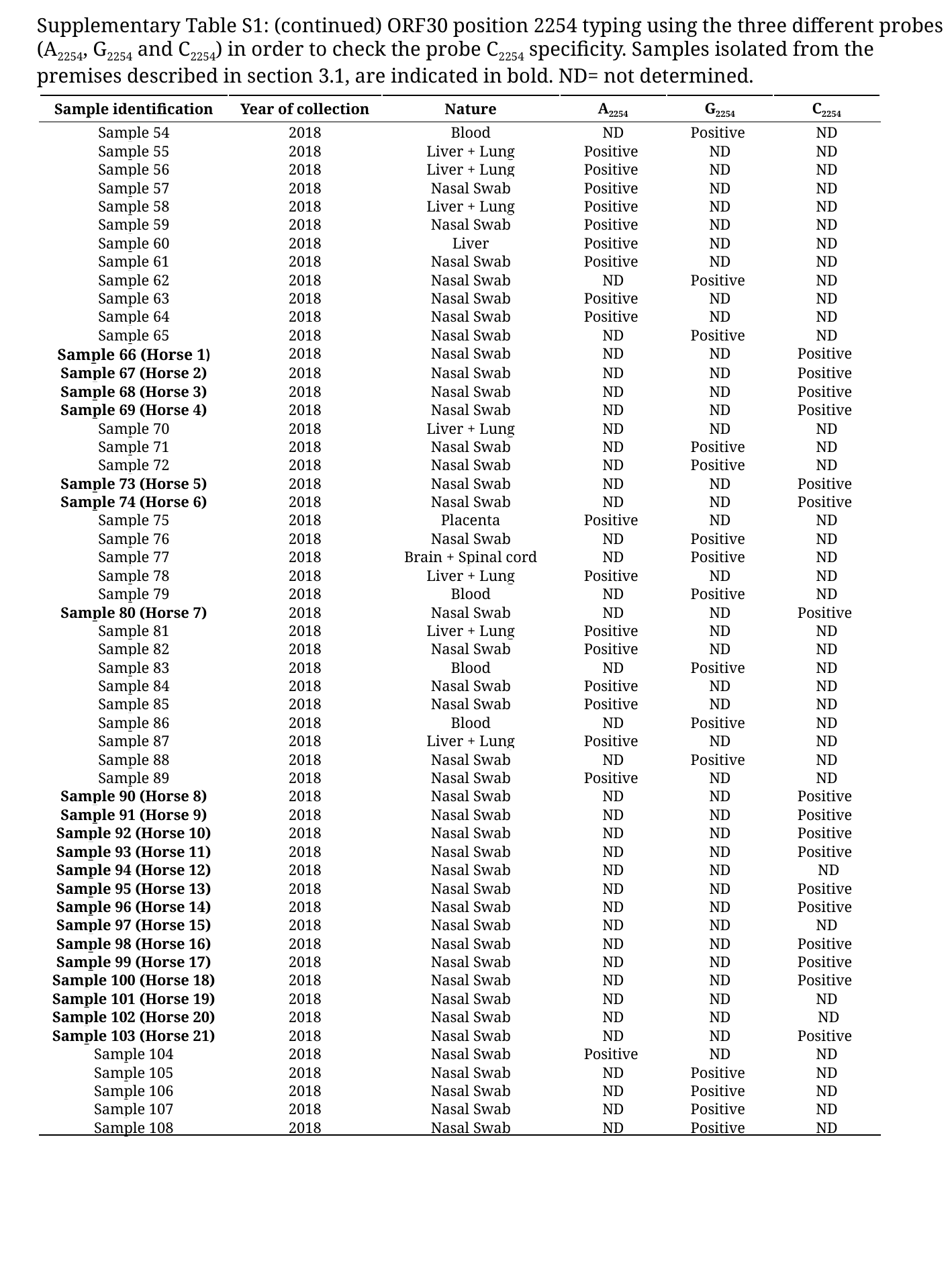

Supplementary Table S1: (continued) ORF30 position 2254 typing using the three different probes (A2254, G2254 and C2254) in order to check the probe C2254 specificity. Samples isolated from the premises described in section 3.1, are indicated in bold. ND= not determined.
| Sample identification | Year of collection | Nature | A2254 | G2254 | C2254 |
| --- | --- | --- | --- | --- | --- |
| Sample 54 | 2018 | Blood | ND | Positive | ND |
| Sample 55 | 2018 | Liver + Lung | Positive | ND | ND |
| Sample 56 | 2018 | Liver + Lung | Positive | ND | ND |
| Sample 57 | 2018 | Nasal Swab | Positive | ND | ND |
| Sample 58 | 2018 | Liver + Lung | Positive | ND | ND |
| Sample 59 | 2018 | Nasal Swab | Positive | ND | ND |
| Sample 60 | 2018 | Liver | Positive | ND | ND |
| Sample 61 | 2018 | Nasal Swab | Positive | ND | ND |
| Sample 62 | 2018 | Nasal Swab | ND | Positive | ND |
| Sample 63 | 2018 | Nasal Swab | Positive | ND | ND |
| Sample 64 | 2018 | Nasal Swab | Positive | ND | ND |
| Sample 65 | 2018 | Nasal Swab | ND | Positive | ND |
| Sample 66 (Horse 1) | 2018 | Nasal Swab | ND | ND | Positive |
| Sample 67 (Horse 2) | 2018 | Nasal Swab | ND | ND | Positive |
| Sample 68 (Horse 3) | 2018 | Nasal Swab | ND | ND | Positive |
| Sample 69 (Horse 4) | 2018 | Nasal Swab | ND | ND | Positive |
| Sample 70 | 2018 | Liver + Lung | ND | ND | ND |
| Sample 71 | 2018 | Nasal Swab | ND | Positive | ND |
| Sample 72 | 2018 | Nasal Swab | ND | Positive | ND |
| Sample 73 (Horse 5) | 2018 | Nasal Swab | ND | ND | Positive |
| Sample 74 (Horse 6) | 2018 | Nasal Swab | ND | ND | Positive |
| Sample 75 | 2018 | Placenta | Positive | ND | ND |
| Sample 76 | 2018 | Nasal Swab | ND | Positive | ND |
| Sample 77 | 2018 | Brain + Spinal cord | ND | Positive | ND |
| Sample 78 | 2018 | Liver + Lung | Positive | ND | ND |
| Sample 79 | 2018 | Blood | ND | Positive | ND |
| Sample 80 (Horse 7) | 2018 | Nasal Swab | ND | ND | Positive |
| Sample 81 | 2018 | Liver + Lung | Positive | ND | ND |
| Sample 82 | 2018 | Nasal Swab | Positive | ND | ND |
| Sample 83 | 2018 | Blood | ND | Positive | ND |
| Sample 84 | 2018 | Nasal Swab | Positive | ND | ND |
| Sample 85 | 2018 | Nasal Swab | Positive | ND | ND |
| Sample 86 | 2018 | Blood | ND | Positive | ND |
| Sample 87 | 2018 | Liver + Lung | Positive | ND | ND |
| Sample 88 | 2018 | Nasal Swab | ND | Positive | ND |
| Sample 89 | 2018 | Nasal Swab | Positive | ND | ND |
| Sample 90 (Horse 8) | 2018 | Nasal Swab | ND | ND | Positive |
| Sample 91 (Horse 9) | 2018 | Nasal Swab | ND | ND | Positive |
| Sample 92 (Horse 10) | 2018 | Nasal Swab | ND | ND | Positive |
| Sample 93 (Horse 11) | 2018 | Nasal Swab | ND | ND | Positive |
| Sample 94 (Horse 12) | 2018 | Nasal Swab | ND | ND | ND |
| Sample 95 (Horse 13) | 2018 | Nasal Swab | ND | ND | Positive |
| Sample 96 (Horse 14) | 2018 | Nasal Swab | ND | ND | Positive |
| Sample 97 (Horse 15) | 2018 | Nasal Swab | ND | ND | ND |
| Sample 98 (Horse 16) | 2018 | Nasal Swab | ND | ND | Positive |
| Sample 99 (Horse 17) | 2018 | Nasal Swab | ND | ND | Positive |
| Sample 100 (Horse 18) | 2018 | Nasal Swab | ND | ND | Positive |
| Sample 101 (Horse 19) | 2018 | Nasal Swab | ND | ND | ND |
| Sample 102 (Horse 20) | 2018 | Nasal Swab | ND | ND | ND |
| Sample 103 (Horse 21) | 2018 | Nasal Swab | ND | ND | Positive |
| Sample 104 | 2018 | Nasal Swab | Positive | ND | ND |
| Sample 105 | 2018 | Nasal Swab | ND | Positive | ND |
| Sample 106 | 2018 | Nasal Swab | ND | Positive | ND |
| Sample 107 | 2018 | Nasal Swab | ND | Positive | ND |
| Sample 108 | 2018 | Nasal Swab | ND | Positive | ND |

## Slide 3
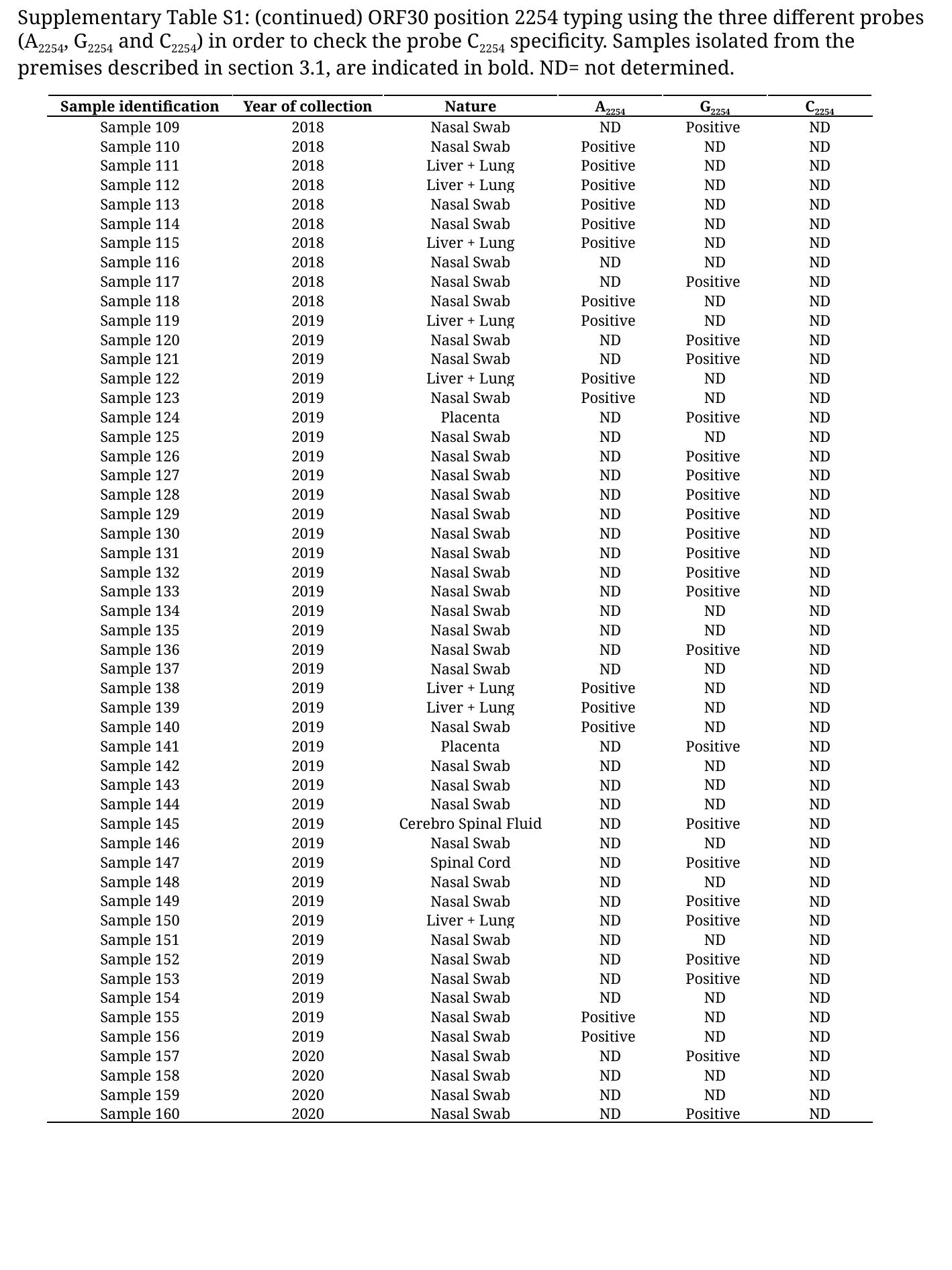

Supplementary Table S1: (continued) ORF30 position 2254 typing using the three different probes (A2254, G2254 and C2254) in order to check the probe C2254 specificity. Samples isolated from the premises described in section 3.1, are indicated in bold. ND= not determined.
| Sample identification | Year of collection | Nature | A2254 | G2254 | C2254 |
| --- | --- | --- | --- | --- | --- |
| Sample 109 | 2018 | Nasal Swab | ND | Positive | ND |
| Sample 110 | 2018 | Nasal Swab | Positive | ND | ND |
| Sample 111 | 2018 | Liver + Lung | Positive | ND | ND |
| Sample 112 | 2018 | Liver + Lung | Positive | ND | ND |
| Sample 113 | 2018 | Nasal Swab | Positive | ND | ND |
| Sample 114 | 2018 | Nasal Swab | Positive | ND | ND |
| Sample 115 | 2018 | Liver + Lung | Positive | ND | ND |
| Sample 116 | 2018 | Nasal Swab | ND | ND | ND |
| Sample 117 | 2018 | Nasal Swab | ND | Positive | ND |
| Sample 118 | 2018 | Nasal Swab | Positive | ND | ND |
| Sample 119 | 2019 | Liver + Lung | Positive | ND | ND |
| Sample 120 | 2019 | Nasal Swab | ND | Positive | ND |
| Sample 121 | 2019 | Nasal Swab | ND | Positive | ND |
| Sample 122 | 2019 | Liver + Lung | Positive | ND | ND |
| Sample 123 | 2019 | Nasal Swab | Positive | ND | ND |
| Sample 124 | 2019 | Placenta | ND | Positive | ND |
| Sample 125 | 2019 | Nasal Swab | ND | ND | ND |
| Sample 126 | 2019 | Nasal Swab | ND | Positive | ND |
| Sample 127 | 2019 | Nasal Swab | ND | Positive | ND |
| Sample 128 | 2019 | Nasal Swab | ND | Positive | ND |
| Sample 129 | 2019 | Nasal Swab | ND | Positive | ND |
| Sample 130 | 2019 | Nasal Swab | ND | Positive | ND |
| Sample 131 | 2019 | Nasal Swab | ND | Positive | ND |
| Sample 132 | 2019 | Nasal Swab | ND | Positive | ND |
| Sample 133 | 2019 | Nasal Swab | ND | Positive | ND |
| Sample 134 | 2019 | Nasal Swab | ND | ND | ND |
| Sample 135 | 2019 | Nasal Swab | ND | ND | ND |
| Sample 136 | 2019 | Nasal Swab | ND | Positive | ND |
| Sample 137 | 2019 | Nasal Swab | ND | ND | ND |
| Sample 138 | 2019 | Liver + Lung | Positive | ND | ND |
| Sample 139 | 2019 | Liver + Lung | Positive | ND | ND |
| Sample 140 | 2019 | Nasal Swab | Positive | ND | ND |
| Sample 141 | 2019 | Placenta | ND | Positive | ND |
| Sample 142 | 2019 | Nasal Swab | ND | ND | ND |
| Sample 143 | 2019 | Nasal Swab | ND | ND | ND |
| Sample 144 | 2019 | Nasal Swab | ND | ND | ND |
| Sample 145 | 2019 | Cerebro Spinal Fluid | ND | Positive | ND |
| Sample 146 | 2019 | Nasal Swab | ND | ND | ND |
| Sample 147 | 2019 | Spinal Cord | ND | Positive | ND |
| Sample 148 | 2019 | Nasal Swab | ND | ND | ND |
| Sample 149 | 2019 | Nasal Swab | ND | Positive | ND |
| Sample 150 | 2019 | Liver + Lung | ND | Positive | ND |
| Sample 151 | 2019 | Nasal Swab | ND | ND | ND |
| Sample 152 | 2019 | Nasal Swab | ND | Positive | ND |
| Sample 153 | 2019 | Nasal Swab | ND | Positive | ND |
| Sample 154 | 2019 | Nasal Swab | ND | ND | ND |
| Sample 155 | 2019 | Nasal Swab | Positive | ND | ND |
| Sample 156 | 2019 | Nasal Swab | Positive | ND | ND |
| Sample 157 | 2020 | Nasal Swab | ND | Positive | ND |
| Sample 158 | 2020 | Nasal Swab | ND | ND | ND |
| Sample 159 | 2020 | Nasal Swab | ND | ND | ND |
| Sample 160 | 2020 | Nasal Swab | ND | Positive | ND |

## Slide 4
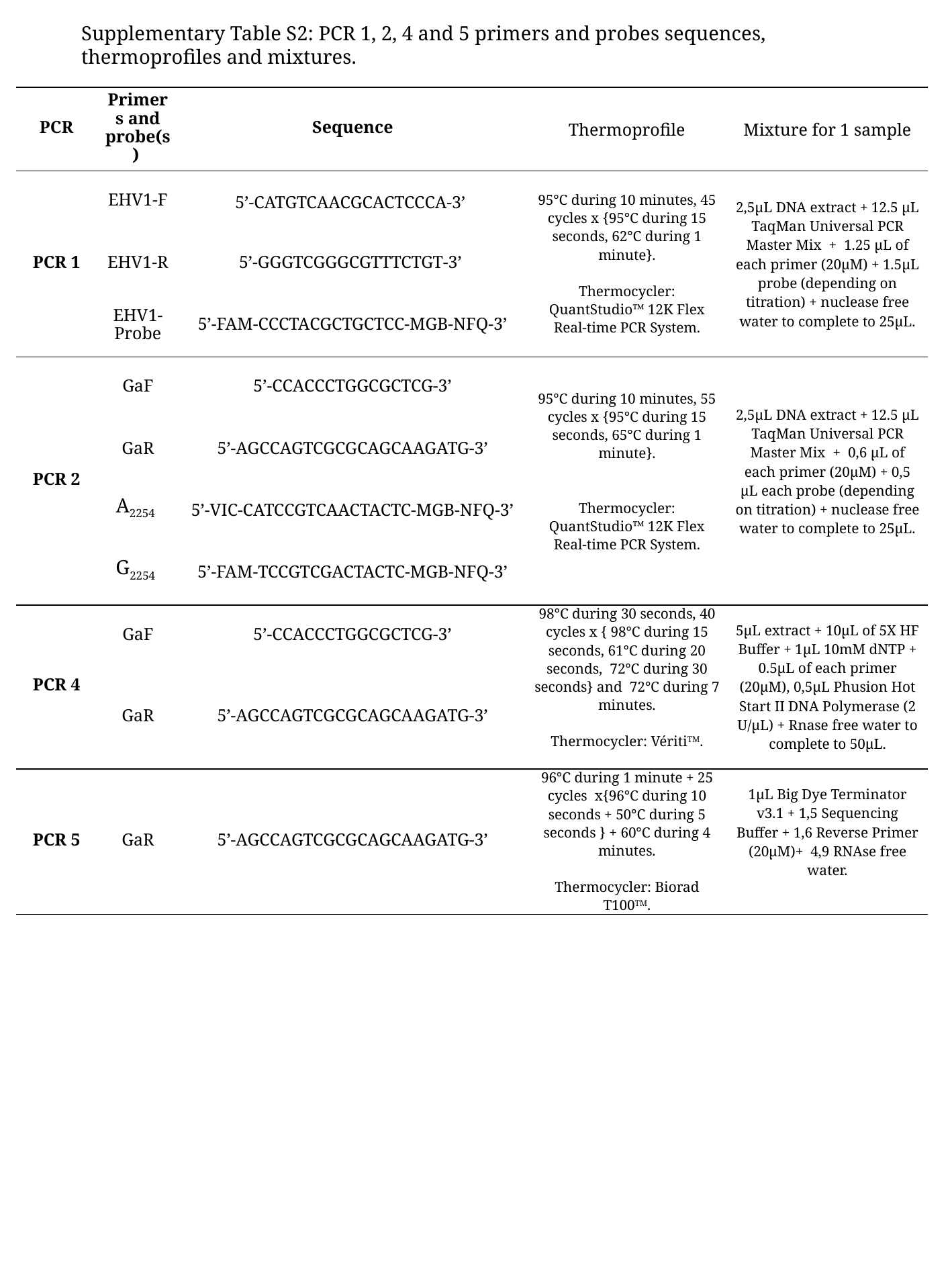

Supplementary Table S2: PCR 1, 2, 4 and 5 primers and probes sequences, thermoprofiles and mixtures.
| PCR | Primers and probe(s) | Sequence | Thermoprofile | Mixture for 1 sample |
| --- | --- | --- | --- | --- |
| PCR 1 | EHV1-F | 5’-CATGTCAACGCACTCCCA-3’ | 95°C during 10 minutes, 45 cycles x {95°C during 15 seconds, 62°C during 1 minute}. Thermocycler: QuantStudio™ 12K Flex Real-time PCR System. | 2,5µL DNA extract + 12.5 μL TaqMan Universal PCR Master Mix + 1.25 μL of each primer (20µM) + 1.5µL probe (depending on titration) + nuclease free water to complete to 25µL. |
| | EHV1-R | 5’-GGGTCGGGCGTTTCTGT-3’ | | |
| | EHV1-Probe | 5’-FAM-CCCTACGCTGCTCC-MGB-NFQ-3’ | | |
| PCR 2 | GaF | 5’-CCACCCTGGCGCTCG-3’ | 95°C during 10 minutes, 55 cycles x {95°C during 15 seconds, 65°C during 1 minute}. Thermocycler: QuantStudio™ 12K Flex Real-time PCR System. | 2,5µL DNA extract + 12.5 μL TaqMan Universal PCR Master Mix + 0,6 μL of each primer (20µM) + 0,5 µL each probe (depending on titration) + nuclease free water to complete to 25µL. |
| | GaR | 5’-AGCCAGTCGCGCAGCAAGATG-3’ | | |
| | A2254 | 5’-VIC-CATCCGTCAACTACTC-MGB-NFQ-3’ | | |
| | G2254 | 5’-FAM-TCCGTCGACTACTC-MGB-NFQ-3’ | | |
| PCR 4 | GaF | 5’-CCACCCTGGCGCTCG-3’ | 98°C during 30 seconds, 40 cycles x { 98°C during 15 seconds, 61°C during 20 seconds, 72°C during 30 seconds} and 72°C during 7 minutes. Thermocycler: VéritiTM. | 5µL extract + 10µL of 5X HF Buffer + 1µL 10mM dNTP + 0.5µL of each primer (20µM), 0,5µL Phusion Hot Start II DNA Polymerase (2 U/µL) + Rnase free water to complete to 50µL. |
| | GaR | 5’-AGCCAGTCGCGCAGCAAGATG-3’ | | |
| PCR 5 | GaR | 5’-AGCCAGTCGCGCAGCAAGATG-3’ | 96°C during 1 minute + 25 cycles x{96°C during 10 seconds + 50°C during 5 seconds } + 60°C during 4 minutes. Thermocycler: Biorad T100TM. | 1µL Big Dye Terminator v3.1 + 1,5 Sequencing Buffer + 1,6 Reverse Primer (20µM)+ 4,9 RNAse free water. |

## Slide 5
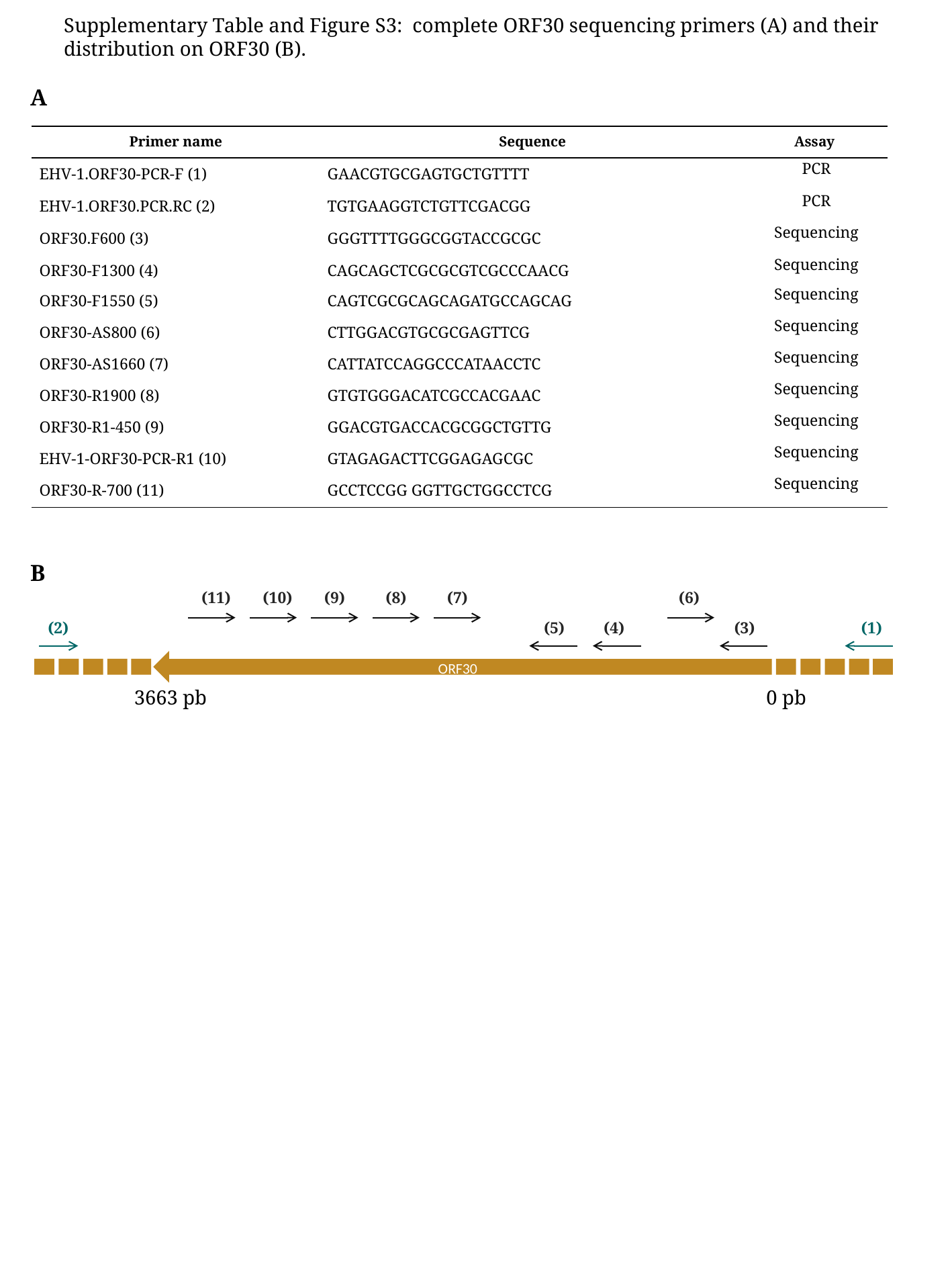

Supplementary Table and Figure S3: complete ORF30 sequencing primers (A) and their distribution on ORF30 (B).
A
| Primer name | Sequence | Assay |
| --- | --- | --- |
| EHV-1.ORF30-PCR-F (1) | GAACGTGCGAGTGCTGTTTT | PCR |
| EHV-1.ORF30.PCR.RC (2) | TGTGAAGGTCTGTTCGACGG | PCR |
| ORF30.F600 (3) | GGGTTTTGGGCGGTACCGCGC | Sequencing |
| ORF30-F1300 (4) | CAGCAGCTCGCGCGTCGCCCAACG | Sequencing |
| ORF30-F1550 (5) | CAGTCGCGCAGCAGATGCCAGCAG | Sequencing |
| ORF30-AS800 (6) | CTTGGACGTGCGCGAGTTCG | Sequencing |
| ORF30-AS1660 (7) | CATTATCCAGGCCCATAACCTC | Sequencing |
| ORF30-R1900 (8) | GTGTGGGACATCGCCACGAAC | Sequencing |
| ORF30-R1-450 (9) | GGACGTGACCACGCGGCTGTTG | Sequencing |
| EHV-1-ORF30-PCR-R1 (10) | GTAGAGACTTCGGAGAGCGC | Sequencing |
| ORF30-R-700 (11) | GCCTCCGG GGTTGCTGGCCTCG | Sequencing |
B
(11)
(10)
(9)
(8)
(7)
(6)
(2)
(5)
(4)
(3)
(1)
ORF30
3663 pb
0 pb

## Slide 6
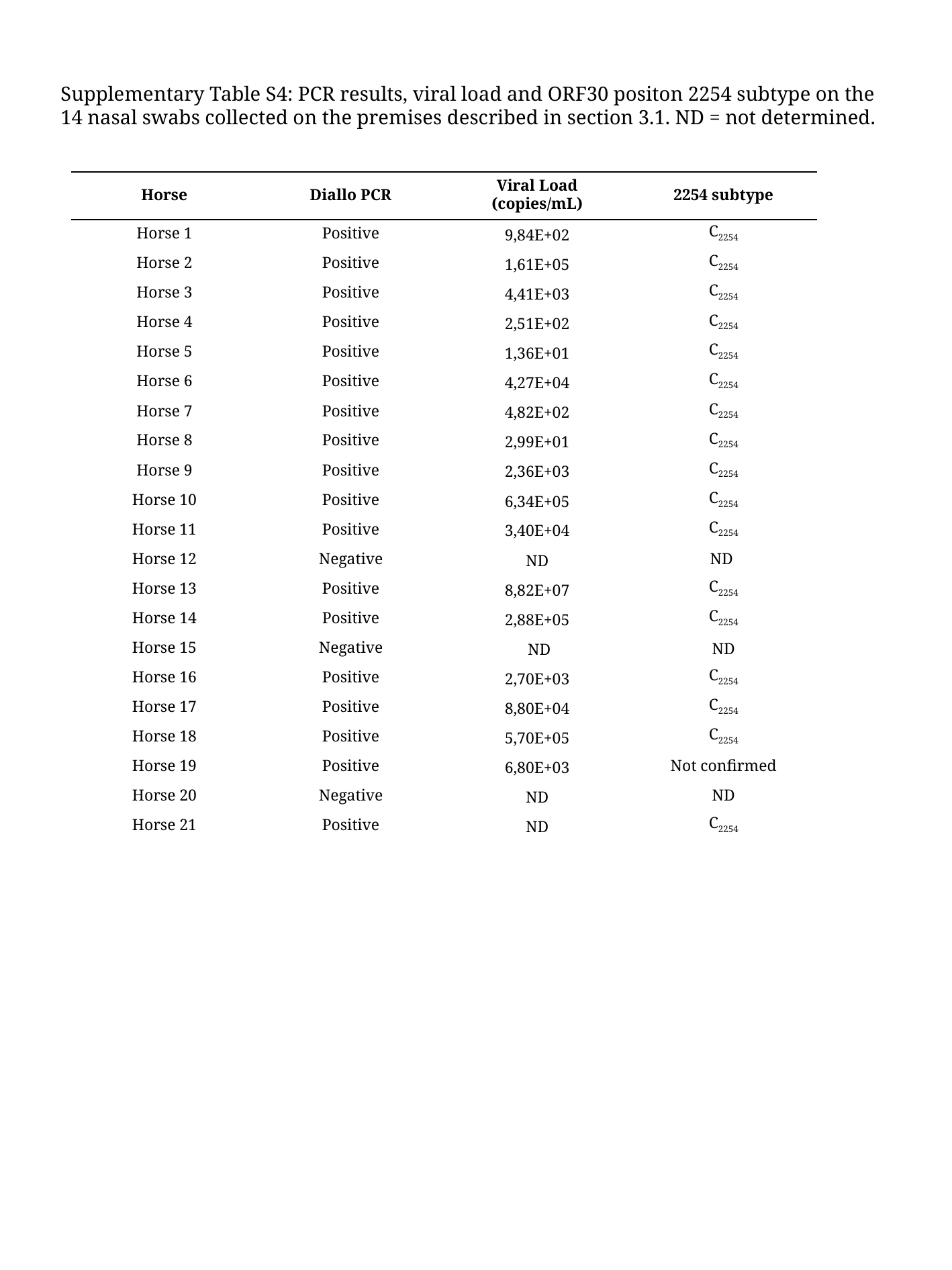

Supplementary Table S4: PCR results, viral load and ORF30 positon 2254 subtype on the 14 nasal swabs collected on the premises described in section 3.1. ND = not determined.
| Horse | Diallo PCR | Viral Load (copies/mL) | 2254 subtype |
| --- | --- | --- | --- |
| Horse 1 | Positive | 9,84E+02 | C2254 |
| Horse 2 | Positive | 1,61E+05 | C2254 |
| Horse 3 | Positive | 4,41E+03 | C2254 |
| Horse 4 | Positive | 2,51E+02 | C2254 |
| Horse 5 | Positive | 1,36E+01 | C2254 |
| Horse 6 | Positive | 4,27E+04 | C2254 |
| Horse 7 | Positive | 4,82E+02 | C2254 |
| Horse 8 | Positive | 2,99E+01 | C2254 |
| Horse 9 | Positive | 2,36E+03 | C2254 |
| Horse 10 | Positive | 6,34E+05 | C2254 |
| Horse 11 | Positive | 3,40E+04 | C2254 |
| Horse 12 | Negative | ND | ND |
| Horse 13 | Positive | 8,82E+07 | C2254 |
| Horse 14 | Positive | 2,88E+05 | C2254 |
| Horse 15 | Negative | ND | ND |
| Horse 16 | Positive | 2,70E+03 | C2254 |
| Horse 17 | Positive | 8,80E+04 | C2254 |
| Horse 18 | Positive | 5,70E+05 | C2254 |
| Horse 19 | Positive | 6,80E+03 | Not confirmed |
| Horse 20 | Negative | ND | ND |
| Horse 21 | Positive | ND | C2254 |

## Slide 7
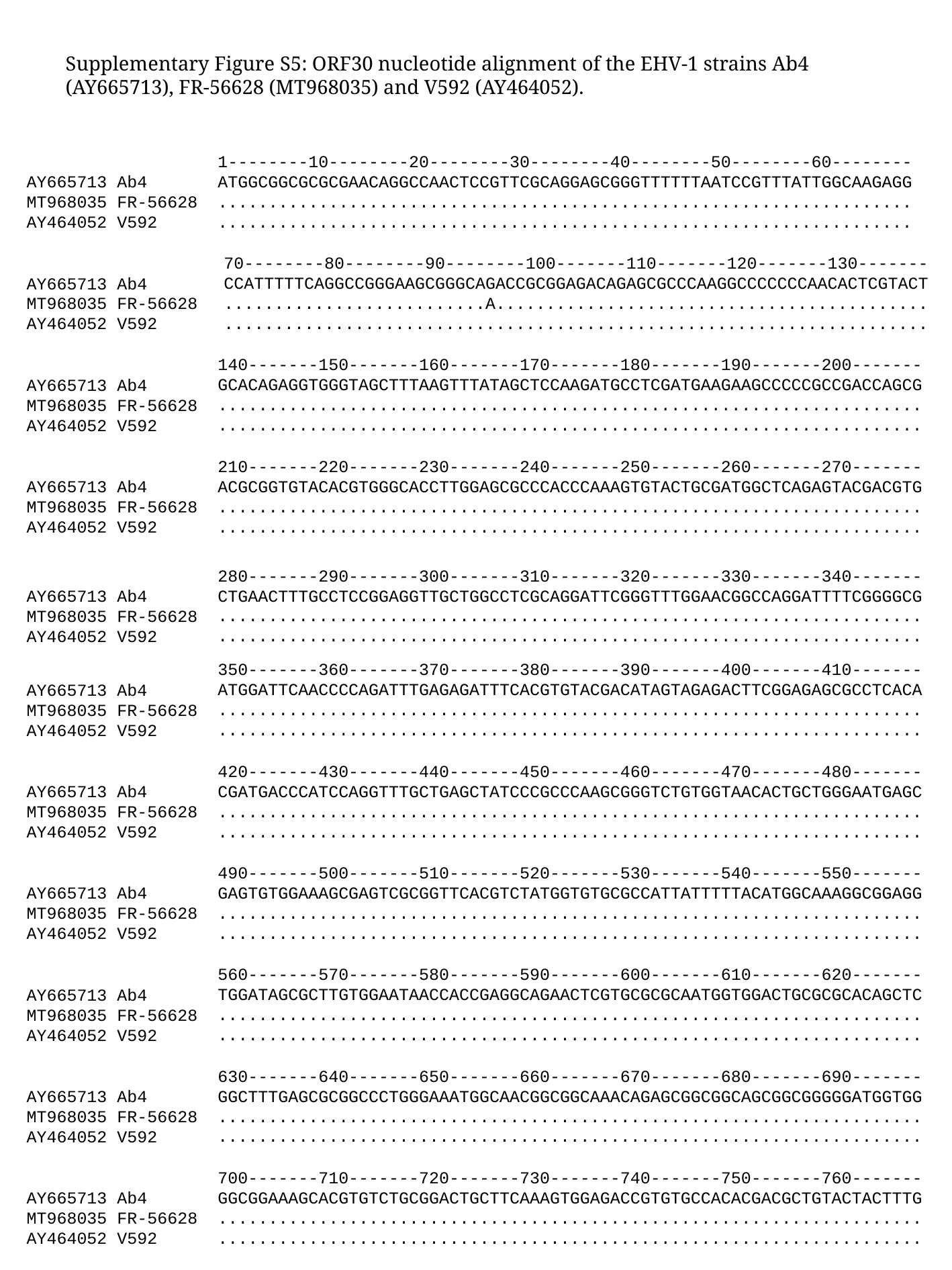

Supplementary Figure S5: ORF30 nucleotide alignment of the EHV-1 strains Ab4 (AY665713), FR-56628 (MT968035) and V592 (AY464052).
1--------10--------20--------30--------40--------50--------60--------
ATGGCGGCGCGCGAACAGGCCAACTCCGTTCGCAGGAGCGGGTTTTTTAATCCGTTTATTGGCAAGAGG
.....................................................................
.....................................................................
AY665713 Ab4
MT968035 FR-56628
AY464052 V592
70--------80--------90--------100-------110-------120-------130-------
CCATTTTTCAGGCCGGGAAGCGGGCAGACCGCGGAGACAGAGCGCCCAAGGCCCCCCCAACACTCGTACT
..........................A...........................................
......................................................................
AY665713 Ab4
MT968035 FR-56628
AY464052 V592
140-------150-------160-------170-------180-------190-------200-------
GCACAGAGGTGGGTAGCTTTAAGTTTATAGCTCCAAGATGCCTCGATGAAGAAGCCCCCGCCGACCAGCG
......................................................................
......................................................................
AY665713 Ab4
MT968035 FR-56628
AY464052 V592
210-------220-------230-------240-------250-------260-------270-------
ACGCGGTGTACACGTGGGCACCTTGGAGCGCCCACCCAAAGTGTACTGCGATGGCTCAGAGTACGACGTG
......................................................................
......................................................................
AY665713 Ab4
MT968035 FR-56628
AY464052 V592
280-------290-------300-------310-------320-------330-------340-------
CTGAACTTTGCCTCCGGAGGTTGCTGGCCTCGCAGGATTCGGGTTTGGAACGGCCAGGATTTTCGGGGCG
......................................................................
......................................................................
AY665713 Ab4
MT968035 FR-56628
AY464052 V592
350-------360-------370-------380-------390-------400-------410-------
ATGGATTCAACCCCAGATTTGAGAGATTTCACGTGTACGACATAGTAGAGACTTCGGAGAGCGCCTCACA
......................................................................
......................................................................
AY665713 Ab4
MT968035 FR-56628
AY464052 V592
420-------430-------440-------450-------460-------470-------480-------
CGATGACCCATCCAGGTTTGCTGAGCTATCCCGCCCAAGCGGGTCTGTGGTAACACTGCTGGGAATGAGC
......................................................................
......................................................................
AY665713 Ab4
MT968035 FR-56628
AY464052 V592
490-------500-------510-------520-------530-------540-------550-------
GAGTGTGGAAAGCGAGTCGCGGTTCACGTCTATGGTGTGCGCCATTATTTTTACATGGCAAAGGCGGAGG
......................................................................
......................................................................
AY665713 Ab4
MT968035 FR-56628
AY464052 V592
560-------570-------580-------590-------600-------610-------620-------
TGGATAGCGCTTGTGGAATAACCACCGAGGCAGAACTCGTGCGCGCAATGGTGGACTGCGCGCACAGCTC
......................................................................
......................................................................
AY665713 Ab4
MT968035 FR-56628
AY464052 V592
630-------640-------650-------660-------670-------680-------690-------
GGCTTTGAGCGCGGCCCTGGGAAATGGCAACGGCGGCAAACAGAGCGGCGGCAGCGGCGGGGGATGGTGG
......................................................................
......................................................................
AY665713 Ab4
MT968035 FR-56628
AY464052 V592
700-------710-------720-------730-------740-------750-------760-------
GGCGGAAAGCACGTGTCTGCGGACTGCTTCAAAGTGGAGACCGTGTGCCACACGACGCTGTACTACTTTG
......................................................................
......................................................................
AY665713 Ab4
MT968035 FR-56628
AY464052 V592

## Slide 8
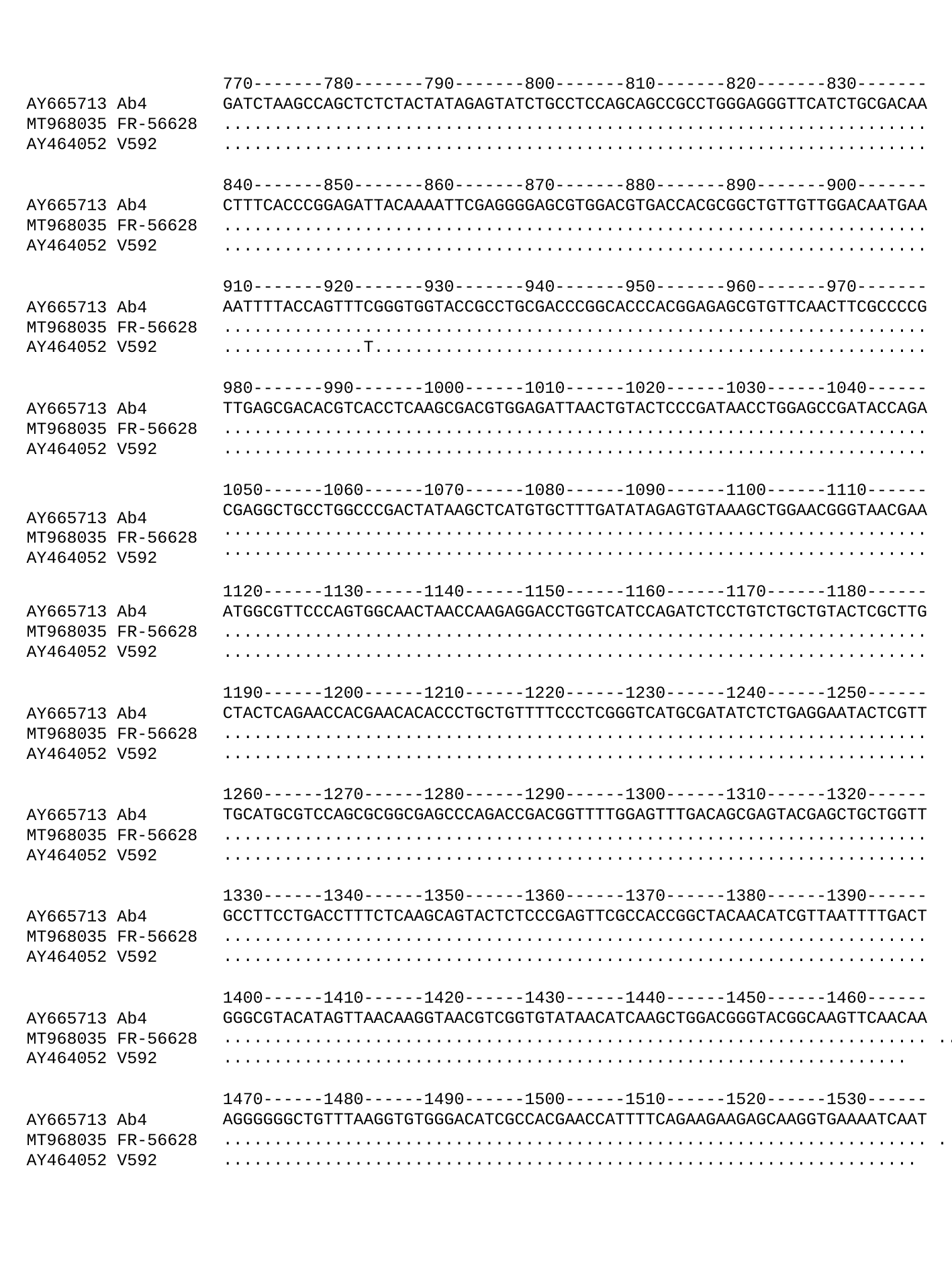

770-------780-------790-------800-------810-------820-------830-------
GATCTAAGCCAGCTCTCTACTATAGAGTATCTGCCTCCAGCAGCCGCCTGGGAGGGTTCATCTGCGACAA
......................................................................
......................................................................
AY665713 Ab4
MT968035 FR-56628
AY464052 V592
840-------850-------860-------870-------880-------890-------900-------
CTTTCACCCGGAGATTACAAAATTCGAGGGGAGCGTGGACGTGACCACGCGGCTGTTGTTGGACAATGAA
......................................................................
......................................................................
AY665713 Ab4
MT968035 FR-56628
AY464052 V592
910-------920-------930-------940-------950-------960-------970-------
AATTTTACCAGTTTCGGGTGGTACCGCCTGCGACCCGGCACCCACGGAGAGCGTGTTCAACTTCGCCCCG
......................................................................
..............T.......................................................
AY665713 Ab4
MT968035 FR-56628
AY464052 V592
980-------990-------1000------1010------1020------1030------1040------
TTGAGCGACACGTCACCTCAAGCGACGTGGAGATTAACTGTACTCCCGATAACCTGGAGCCGATACCAGA
......................................................................
......................................................................
AY665713 Ab4
MT968035 FR-56628
AY464052 V592
1050------1060------1070------1080------1090------1100------1110------
CGAGGCTGCCTGGCCCGACTATAAGCTCATGTGCTTTGATATAGAGTGTAAAGCTGGAACGGGTAACGAA
......................................................................
......................................................................
AY665713 Ab4
MT968035 FR-56628
AY464052 V592
1120------1130------1140------1150------1160------1170------1180------
ATGGCGTTCCCAGTGGCAACTAACCAAGAGGACCTGGTCATCCAGATCTCCTGTCTGCTGTACTCGCTTG
......................................................................
......................................................................
AY665713 Ab4
MT968035 FR-56628
AY464052 V592
1190------1200------1210------1220------1230------1240------1250------
CTACTCAGAACCACGAACACACCCTGCTGTTTTCCCTCGGGTCATGCGATATCTCTGAGGAATACTCGTT
......................................................................
......................................................................
AY665713 Ab4
MT968035 FR-56628
AY464052 V592
1260------1270------1280------1290------1300------1310------1320------
TGCATGCGTCCAGCGCGGCGAGCCCAGACCGACGGTTTTGGAGTTTGACAGCGAGTACGAGCTGCTGGTT
......................................................................
......................................................................
AY665713 Ab4
MT968035 FR-56628
AY464052 V592
1330------1340------1350------1360------1370------1380------1390------
GCCTTCCTGACCTTTCTCAAGCAGTACTCTCCCGAGTTCGCCACCGGCTACAACATCGTTAATTTTGACT
......................................................................
......................................................................
AY665713 Ab4
MT968035 FR-56628
AY464052 V592
1400------1410------1420------1430------1440------1450------1460------
GGGCGTACATAGTTAACAAGGTAACGTCGGTGTATAACATCAAGCTGGACGGGTACGGCAAGTTCAACAA
...................................................................... ......................................................................
AY665713 Ab4
MT968035 FR-56628
AY464052 V592
1470------1480------1490------1500------1510------1520------1530------
AGGGGGGCTGTTTAAGGTGTGGGACATCGCCACGAACCATTTTCAGAAGAAGAGCAAGGTGAAAATCAAT
...................................................................... ......................................................................
AY665713 Ab4
MT968035 FR-56628
AY464052 V592

## Slide 9
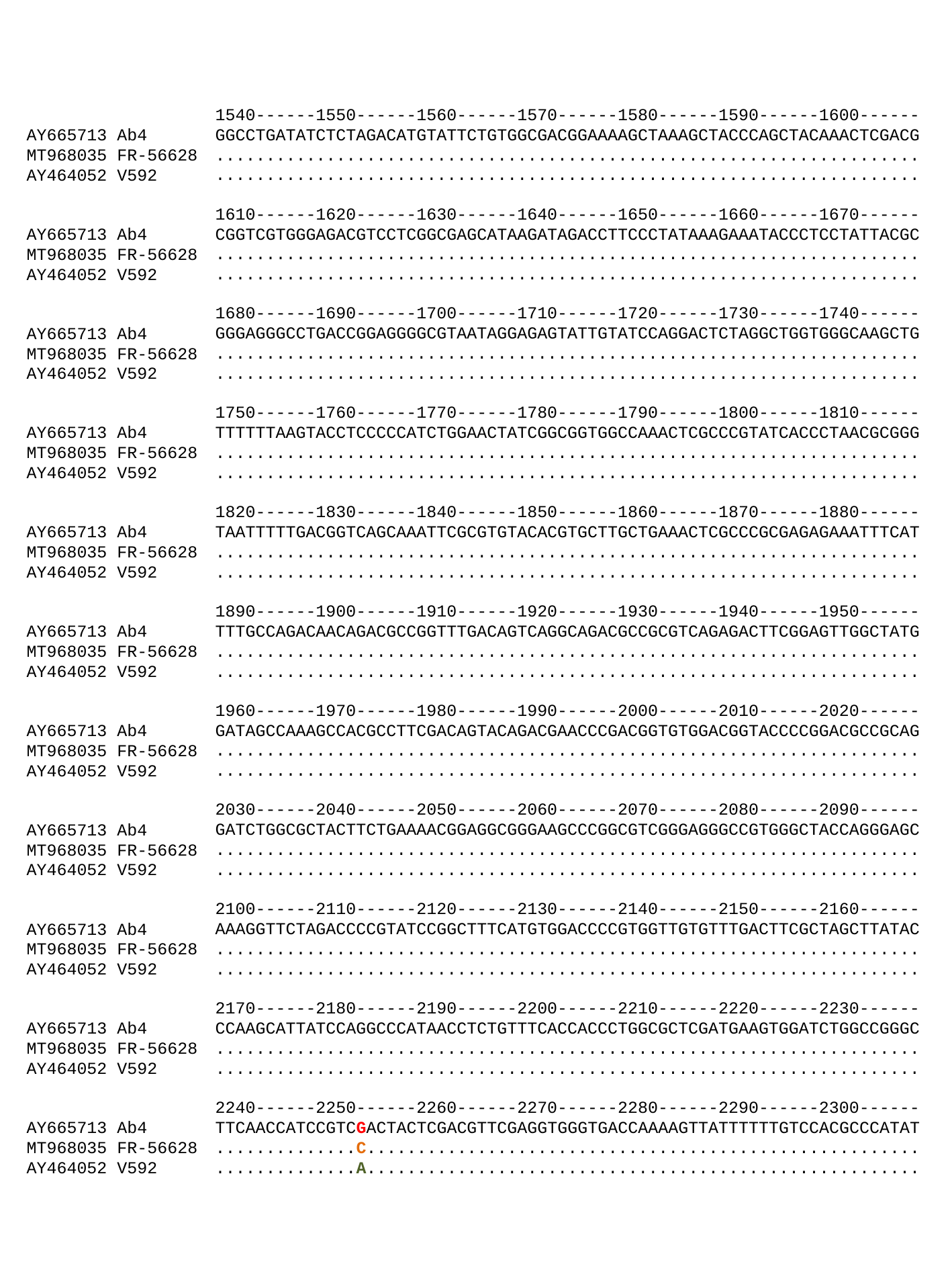

1540------1550------1560------1570------1580------1590------1600------
GGCCTGATATCTCTAGACATGTATTCTGTGGCGACGGAAAAGCTAAAGCTACCCAGCTACAAACTCGACG
......................................................................
......................................................................
AY665713 Ab4
MT968035 FR-56628
AY464052 V592
1610------1620------1630------1640------1650------1660------1670------
CGGTCGTGGGAGACGTCCTCGGCGAGCATAAGATAGACCTTCCCTATAAAGAAATACCCTCCTATTACGC
......................................................................
......................................................................
AY665713 Ab4
MT968035 FR-56628
AY464052 V592
1680------1690------1700------1710------1720------1730------1740------
GGGAGGGCCTGACCGGAGGGGCGTAATAGGAGAGTATTGTATCCAGGACTCTAGGCTGGTGGGCAAGCTG
......................................................................
......................................................................
AY665713 Ab4
MT968035 FR-56628
AY464052 V592
1750------1760------1770------1780------1790------1800------1810------
TTTTTTAAGTACCTCCCCCATCTGGAACTATCGGCGGTGGCCAAACTCGCCCGTATCACCCTAACGCGGG
......................................................................
......................................................................
AY665713 Ab4
MT968035 FR-56628
AY464052 V592
1820------1830------1840------1850------1860------1870------1880------
TAATTTTTGACGGTCAGCAAATTCGCGTGTACACGTGCTTGCTGAAACTCGCCCGCGAGAGAAATTTCAT
......................................................................
......................................................................
AY665713 Ab4
MT968035 FR-56628
AY464052 V592
1890------1900------1910------1920------1930------1940------1950------
TTTGCCAGACAACAGACGCCGGTTTGACAGTCAGGCAGACGCCGCGTCAGAGACTTCGGAGTTGGCTATG
......................................................................
......................................................................
AY665713 Ab4
MT968035 FR-56628
AY464052 V592
1960------1970------1980------1990------2000------2010------2020------
GATAGCCAAAGCCACGCCTTCGACAGTACAGACGAACCCGACGGTGTGGACGGTACCCCGGACGCCGCAG
......................................................................
......................................................................
AY665713 Ab4
MT968035 FR-56628
AY464052 V592
2030------2040------2050------2060------2070------2080------2090------
GATCTGGCGCTACTTCTGAAAACGGAGGCGGGAAGCCCGGCGTCGGGAGGGCCGTGGGCTACCAGGGAGC
......................................................................
......................................................................
AY665713 Ab4
MT968035 FR-56628
AY464052 V592
2100------2110------2120------2130------2140------2150------2160------
AAAGGTTCTAGACCCCGTATCCGGCTTTCATGTGGACCCCGTGGTTGTGTTTGACTTCGCTAGCTTATAC
......................................................................
......................................................................
AY665713 Ab4
MT968035 FR-56628
AY464052 V592
2170------2180------2190------2200------2210------2220------2230------
CCAAGCATTATCCAGGCCCATAACCTCTGTTTCACCACCCTGGCGCTCGATGAAGTGGATCTGGCCGGGC
......................................................................
......................................................................
AY665713 Ab4
MT968035 FR-56628
AY464052 V592
2240------2250------2260------2270------2280------2290------2300------
TTCAACCATCCGTCGACTACTCGACGTTCGAGGTGGGTGACCAAAAGTTATTTTTTGTCCACGCCCATAT
..............C.......................................................
..............A.......................................................
AY665713 Ab4
MT968035 FR-56628
AY464052 V592

## Slide 10
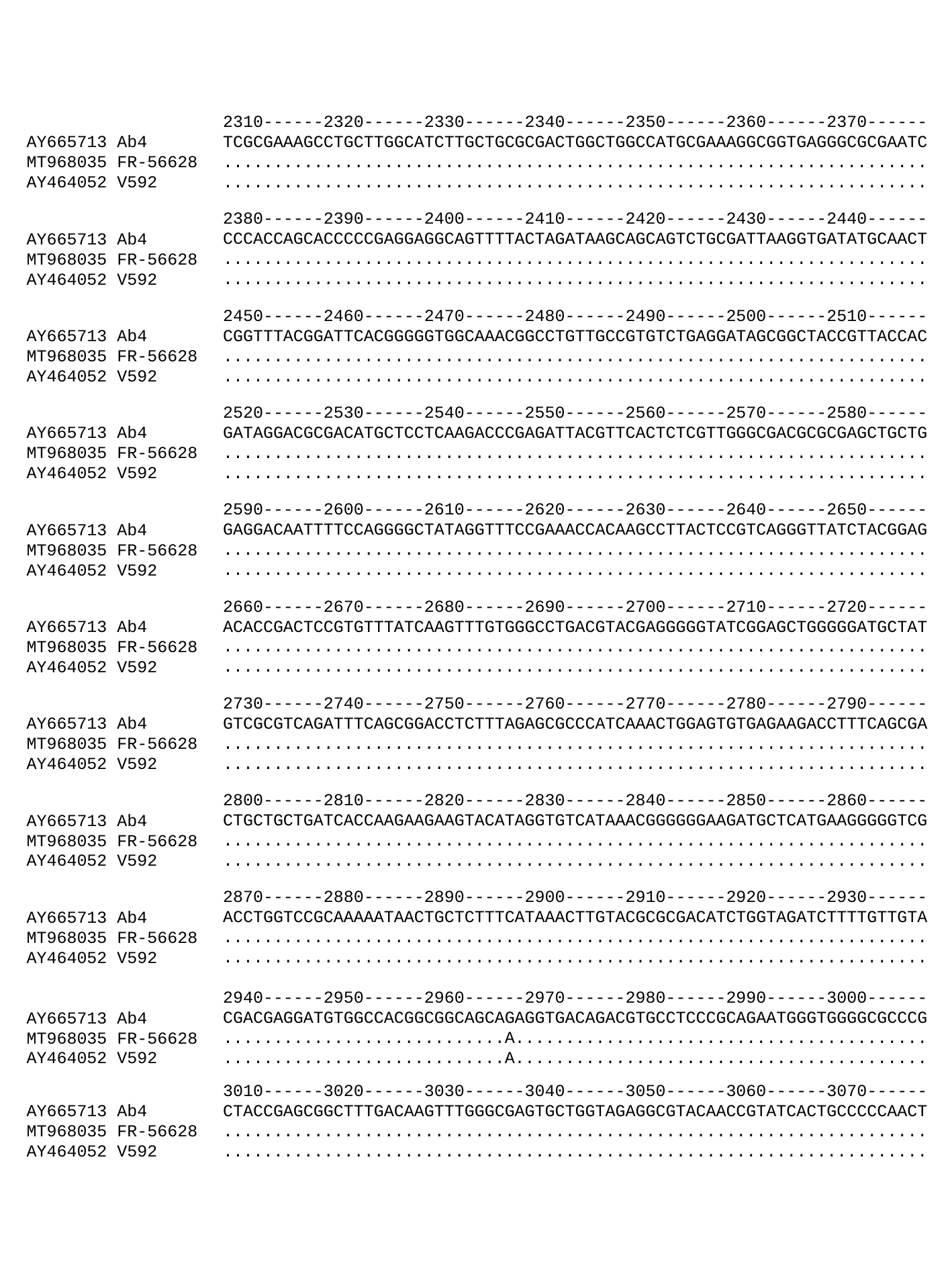

2310------2320------2330------2340------2350------2360------2370------
TCGCGAAAGCCTGCTTGGCATCTTGCTGCGCGACTGGCTGGCCATGCGAAAGGCGGTGAGGGCGCGAATC
......................................................................
......................................................................
AY665713 Ab4
MT968035 FR-56628
AY464052 V592
2380------2390------2400------2410------2420------2430------2440------
CCCACCAGCACCCCCGAGGAGGCAGTTTTACTAGATAAGCAGCAGTCTGCGATTAAGGTGATATGCAACT
......................................................................
......................................................................
AY665713 Ab4
MT968035 FR-56628
AY464052 V592
2450------2460------2470------2480------2490------2500------2510------
CGGTTTACGGATTCACGGGGGTGGCAAACGGCCTGTTGCCGTGTCTGAGGATAGCGGCTACCGTTACCAC
......................................................................
......................................................................
AY665713 Ab4
MT968035 FR-56628
AY464052 V592
2520------2530------2540------2550------2560------2570------2580------
GATAGGACGCGACATGCTCCTCAAGACCCGAGATTACGTTCACTCTCGTTGGGCGACGCGCGAGCTGCTG
......................................................................
......................................................................
AY665713 Ab4
MT968035 FR-56628
AY464052 V592
2590------2600------2610------2620------2630------2640------2650------
GAGGACAATTTTCCAGGGGCTATAGGTTTCCGAAACCACAAGCCTTACTCCGTCAGGGTTATCTACGGAG
......................................................................
......................................................................
AY665713 Ab4
MT968035 FR-56628
AY464052 V592
2660------2670------2680------2690------2700------2710------2720------
ACACCGACTCCGTGTTTATCAAGTTTGTGGGCCTGACGTACGAGGGGGTATCGGAGCTGGGGGATGCTAT
......................................................................
......................................................................
AY665713 Ab4
MT968035 FR-56628
AY464052 V592
2730------2740------2750------2760------2770------2780------2790------ GTCGCGTCAGATTTCAGCGGACCTCTTTAGAGCGCCCATCAAACTGGAGTGTGAGAAGACCTTTCAGCGA
......................................................................
......................................................................
AY665713 Ab4
MT968035 FR-56628
AY464052 V592
2800------2810------2820------2830------2840------2850------2860------ CTGCTGCTGATCACCAAGAAGAAGTACATAGGTGTCATAAACGGGGGGAAGATGCTCATGAAGGGGGTCG
......................................................................
......................................................................
AY665713 Ab4
MT968035 FR-56628
AY464052 V592
2870------2880------2890------2900------2910------2920------2930------ ACCTGGTCCGCAAAAATAACTGCTCTTTCATAAACTTGTACGCGCGACATCTGGTAGATCTTTTGTTGTA
......................................................................
......................................................................
AY665713 Ab4
MT968035 FR-56628
AY464052 V592
2940------2950------2960------2970------2980------2990------3000------ CGACGAGGATGTGGCCACGGCGGCAGCAGAGGTGACAGACGTGCCTCCCGCAGAATGGGTGGGGCGCCCG
............................A.........................................
............................A.........................................
AY665713 Ab4
MT968035 FR-56628
AY464052 V592
3010------3020------3030------3040------3050------3060------3070------ CTACCGAGCGGCTTTGACAAGTTTGGGCGAGTGCTGGTAGAGGCGTACAACCGTATCACTGCCCCCAACT
......................................................................
......................................................................
AY665713 Ab4
MT968035 FR-56628
AY464052 V592

## Slide 11
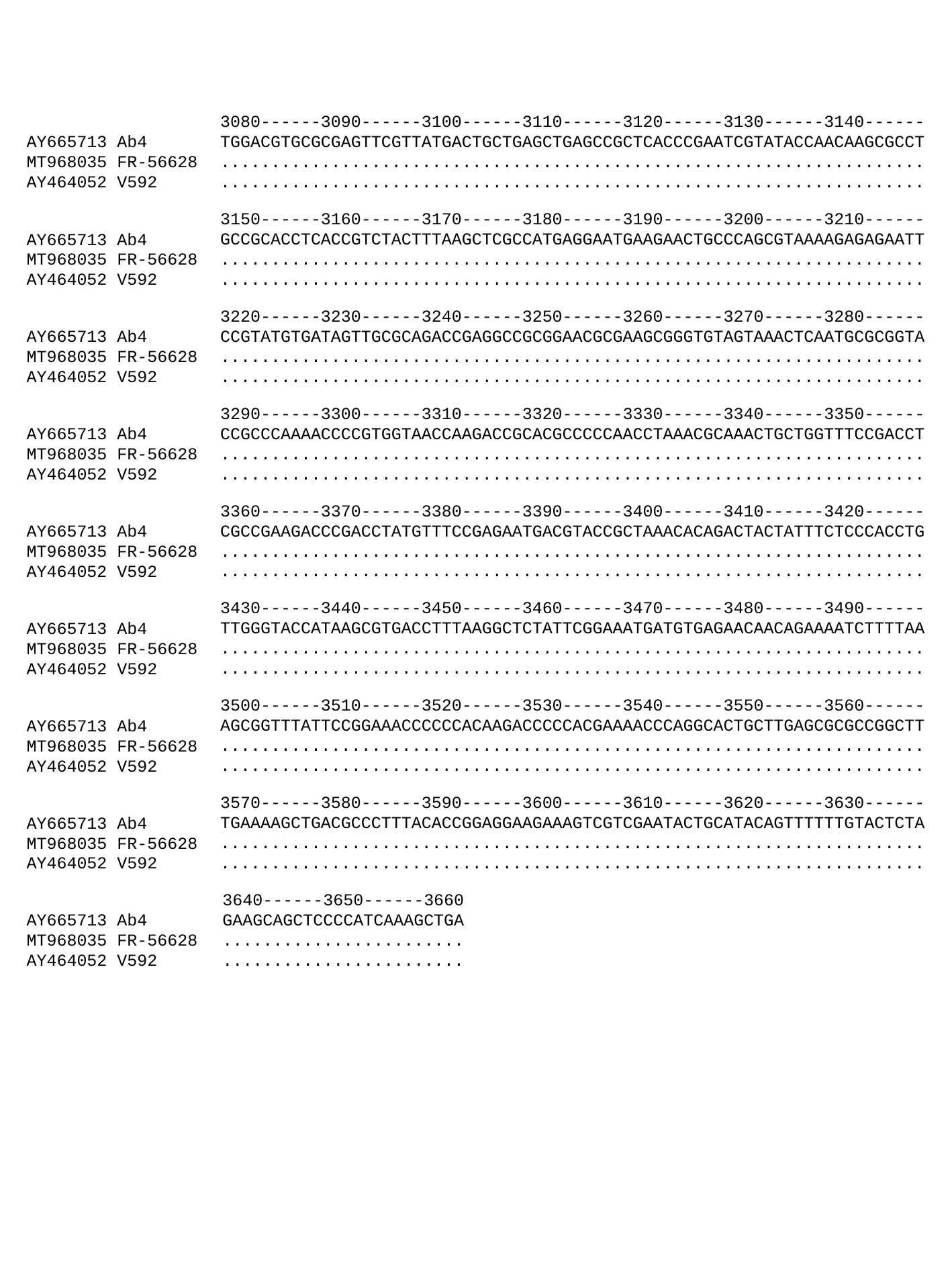

3080------3090------3100------3110------3120------3130------3140------ TGGACGTGCGCGAGTTCGTTATGACTGCTGAGCTGAGCCGCTCACCCGAATCGTATACCAACAAGCGCCT
......................................................................
......................................................................
AY665713 Ab4
MT968035 FR-56628
AY464052 V592
3150------3160------3170------3180------3190------3200------3210------ GCCGCACCTCACCGTCTACTTTAAGCTCGCCATGAGGAATGAAGAACTGCCCAGCGTAAAAGAGAGAATT
......................................................................
......................................................................
AY665713 Ab4
MT968035 FR-56628
AY464052 V592
3220------3230------3240------3250------3260------3270------3280------ CCGTATGTGATAGTTGCGCAGACCGAGGCCGCGGAACGCGAAGCGGGTGTAGTAAACTCAATGCGCGGTA
......................................................................
......................................................................
AY665713 Ab4
MT968035 FR-56628
AY464052 V592
3290------3300------3310------3320------3330------3340------3350------ CCGCCCAAAACCCCGTGGTAACCAAGACCGCACGCCCCCAACCTAAACGCAAACTGCTGGTTTCCGACCT
......................................................................
......................................................................
AY665713 Ab4
MT968035 FR-56628
AY464052 V592
3360------3370------3380------3390------3400------3410------3420------ CGCCGAAGACCCGACCTATGTTTCCGAGAATGACGTACCGCTAAACACAGACTACTATTTCTCCCACCTG
......................................................................
......................................................................
AY665713 Ab4
MT968035 FR-56628
AY464052 V592
3430------3440------3450------3460------3470------3480------3490------ TTGGGTACCATAAGCGTGACCTTTAAGGCTCTATTCGGAAATGATGTGAGAACAACAGAAAATCTTTTAA
......................................................................
......................................................................
AY665713 Ab4
MT968035 FR-56628
AY464052 V592
3500------3510------3520------3530------3540------3550------3560------ AGCGGTTTATTCCGGAAACCCCCCACAAGACCCCCACGAAAACCCAGGCACTGCTTGAGCGCGCCGGCTT
......................................................................
......................................................................
AY665713 Ab4
MT968035 FR-56628
AY464052 V592
3570------3580------3590------3600------3610------3620------3630------ TGAAAAGCTGACGCCCTTTACACCGGAGGAAGAAAGTCGTCGAATACTGCATACAGTTTTTTGTACTCTA
......................................................................
......................................................................
AY665713 Ab4
MT968035 FR-56628
AY464052 V592
3640------3650------3660
GAAGCAGCTCCCCATCAAAGCTGA
........................
........................
AY665713 Ab4
MT968035 FR-56628
AY464052 V592

## Slide 12
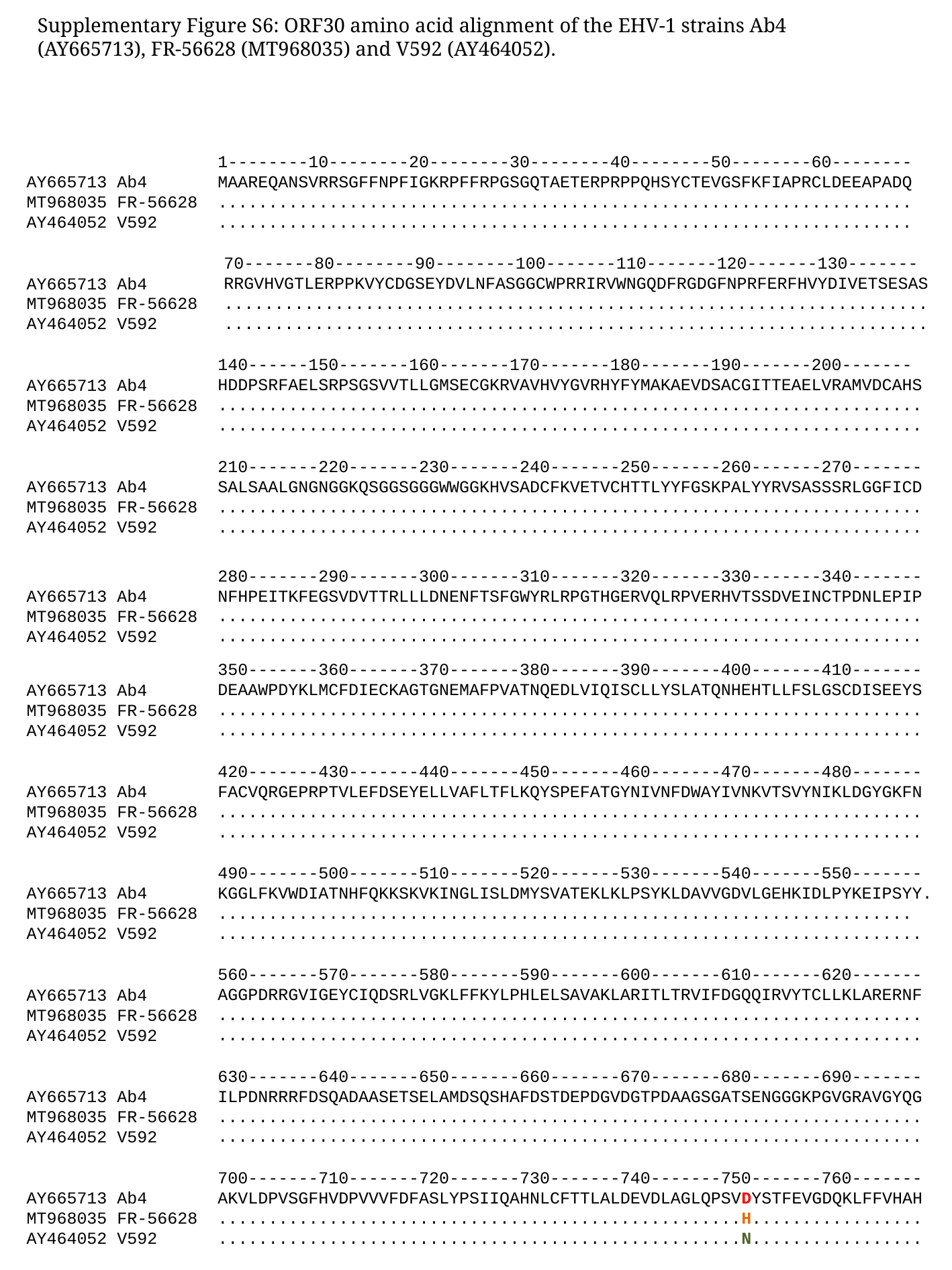

Supplementary Figure S6: ORF30 amino acid alignment of the EHV-1 strains Ab4 (AY665713), FR-56628 (MT968035) and V592 (AY464052).
1--------10--------20--------30--------40--------50--------60--------
MAAREQANSVRRSGFFNPFIGKRPFFRPGSGQTAETERPRPPQHSYCTEVGSFKFIAPRCLDEEAPADQ
.....................................................................
.....................................................................
AY665713 Ab4
MT968035 FR-56628
AY464052 V592
70-------80--------90--------100-------110-------120-------130-------
RRGVHVGTLERPPKVYCDGSEYDVLNFASGGCWPRRIRVWNGQDFRGDGFNPRFERFHVYDIVETSESAS
......................................................................
......................................................................
AY665713 Ab4
MT968035 FR-56628
AY464052 V592
140------150-------160-------170-------180-------190-------200-------
HDDPSRFAELSRPSGSVVTLLGMSECGKRVAVHVYGVRHYFYMAKAEVDSACGITTEAELVRAMVDCAHS
......................................................................
......................................................................
AY665713 Ab4
MT968035 FR-56628
AY464052 V592
210-------220-------230-------240-------250-------260-------270-------
SALSAALGNGNGGKQSGGSGGGWWGGKHVSADCFKVETVCHTTLYYFGSKPALYYRVSASSSRLGGFICD
......................................................................
......................................................................
AY665713 Ab4
MT968035 FR-56628
AY464052 V592
280-------290-------300-------310-------320-------330-------340-------
NFHPEITKFEGSVDVTTRLLLDNENFTSFGWYRLRPGTHGERVQLRPVERHVTSSDVEINCTPDNLEPIP
......................................................................
......................................................................
AY665713 Ab4
MT968035 FR-56628
AY464052 V592
350-------360-------370-------380-------390-------400-------410-------
DEAAWPDYKLMCFDIECKAGTGNEMAFPVATNQEDLVIQISCLLYSLATQNHEHTLLFSLGSCDISEEYS
......................................................................
......................................................................
AY665713 Ab4
MT968035 FR-56628
AY464052 V592
420-------430-------440-------450-------460-------470-------480-------
FACVQRGEPRPTVLEFDSEYELLVAFLTFLKQYSPEFATGYNIVNFDWAYIVNKVTSVYNIKLDGYGKFN
......................................................................
......................................................................
AY665713 Ab4
MT968035 FR-56628
AY464052 V592
490-------500-------510-------520-------530-------540-------550-------
KGGLFKVWDIATNHFQKKSKVKINGLISLDMYSVATEKLKLPSYKLDAVVGDVLGEHKIDLPYKEIPSYY......................................................................
......................................................................
AY665713 Ab4
MT968035 FR-56628
AY464052 V592
560-------570-------580-------590-------600-------610-------620-------
AGGPDRRGVIGEYCIQDSRLVGKLFFKYLPHLELSAVAKLARITLTRVIFDGQQIRVYTCLLKLARERNF......................................................................
......................................................................
AY665713 Ab4
MT968035 FR-56628
AY464052 V592
630-------640-------650-------660-------670-------680-------690-------
ILPDNRRRFDSQADAASETSELAMDSQSHAFDSTDEPDGVDGTPDAAGSGATSENGGGKPGVGRAVGYQG
......................................................................
......................................................................
AY665713 Ab4
MT968035 FR-56628
AY464052 V592
700-------710-------720-------730-------740-------750-------760-------
AKVLDPVSGFHVDPVVVFDFASLYPSIIQAHNLCFTTLALDEVDLAGLQPSVDYSTFEVGDQKLFFVHAH
....................................................H.................
....................................................N.................
AY665713 Ab4
MT968035 FR-56628
AY464052 V592

## Slide 13
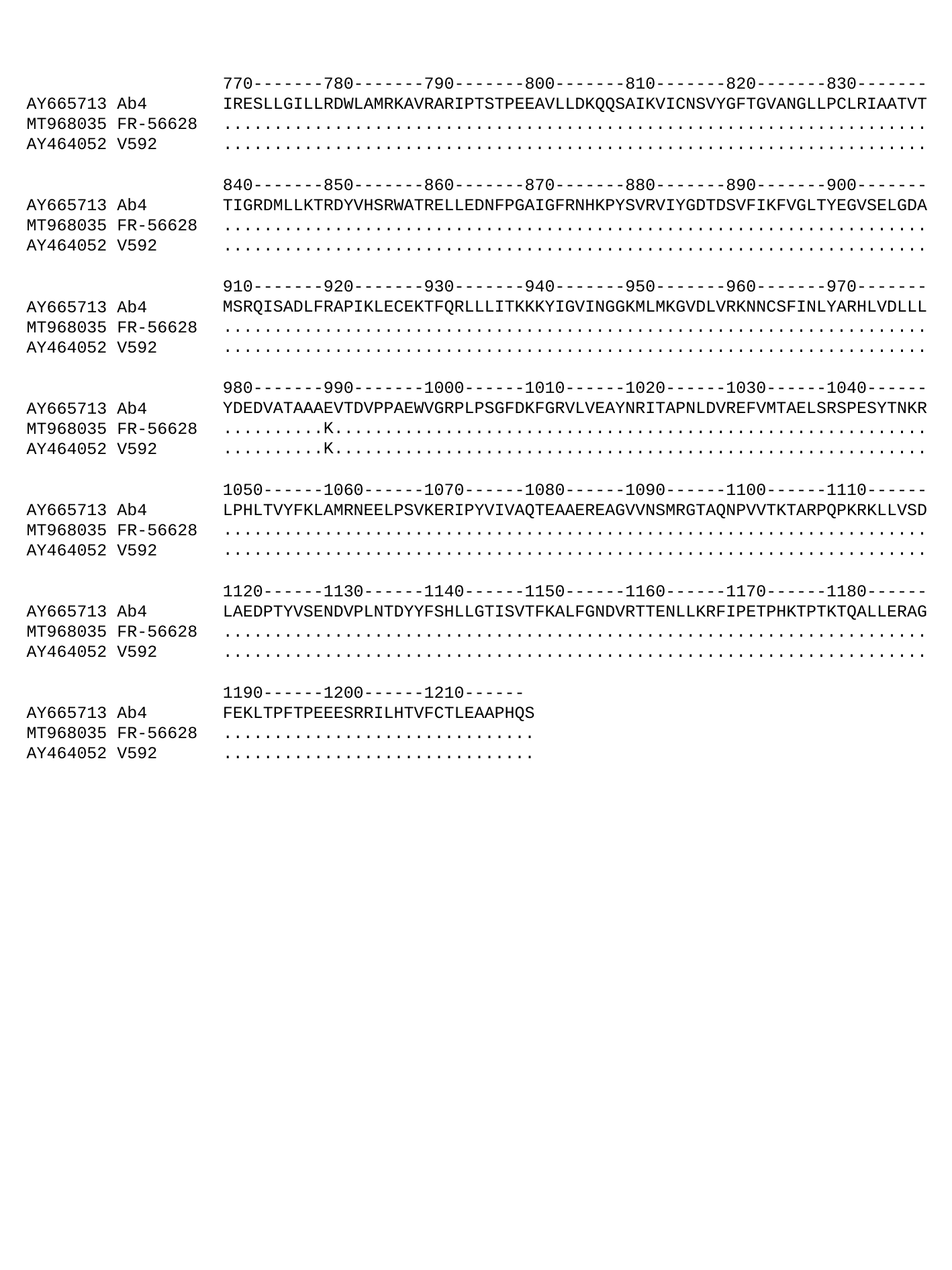

770-------780-------790-------800-------810-------820-------830-------
IRESLLGILLRDWLAMRKAVRARIPTSTPEEAVLLDKQQSAIKVICNSVYGFTGVANGLLPCLRIAATVT
......................................................................
......................................................................
AY665713 Ab4
MT968035 FR-56628
AY464052 V592
840-------850-------860-------870-------880-------890-------900-------
TIGRDMLLKTRDYVHSRWATRELLEDNFPGAIGFRNHKPYSVRVIYGDTDSVFIKFVGLTYEGVSELGDA
......................................................................
......................................................................
AY665713 Ab4
MT968035 FR-56628
AY464052 V592
910-------920-------930-------940-------950-------960-------970-------
MSRQISADLFRAPIKLECEKTFQRLLLITKKKYIGVINGGKMLMKGVDLVRKNNCSFINLYARHLVDLLL
......................................................................
......................................................................
AY665713 Ab4
MT968035 FR-56628
AY464052 V592
980-------990-------1000------1010------1020------1030------1040------
YDEDVATAAAEVTDVPPAEWVGRPLPSGFDKFGRVLVEAYNRITAPNLDVREFVMTAELSRSPESYTNKR
..........K...........................................................
..........K...........................................................
AY665713 Ab4
MT968035 FR-56628
AY464052 V592
1050------1060------1070------1080------1090------1100------1110------
LPHLTVYFKLAMRNEELPSVKERIPYVIVAQTEAAEREAGVVNSMRGTAQNPVVTKTARPQPKRKLLVSD
......................................................................
......................................................................
AY665713 Ab4
MT968035 FR-56628
AY464052 V592
1120------1130------1140------1150------1160------1170------1180------
LAEDPTYVSENDVPLNTDYYFSHLLGTISVTFKALFGNDVRTTENLLKRFIPETPHKTPTKTQALLERAG
......................................................................
......................................................................
AY665713 Ab4
MT968035 FR-56628
AY464052 V592
1190------1200------1210------
FEKLTPFTPEEESRRILHTVFCTLEAAPHQS
...............................
...............................
AY665713 Ab4
MT968035 FR-56628
AY464052 V592

## Slide 14
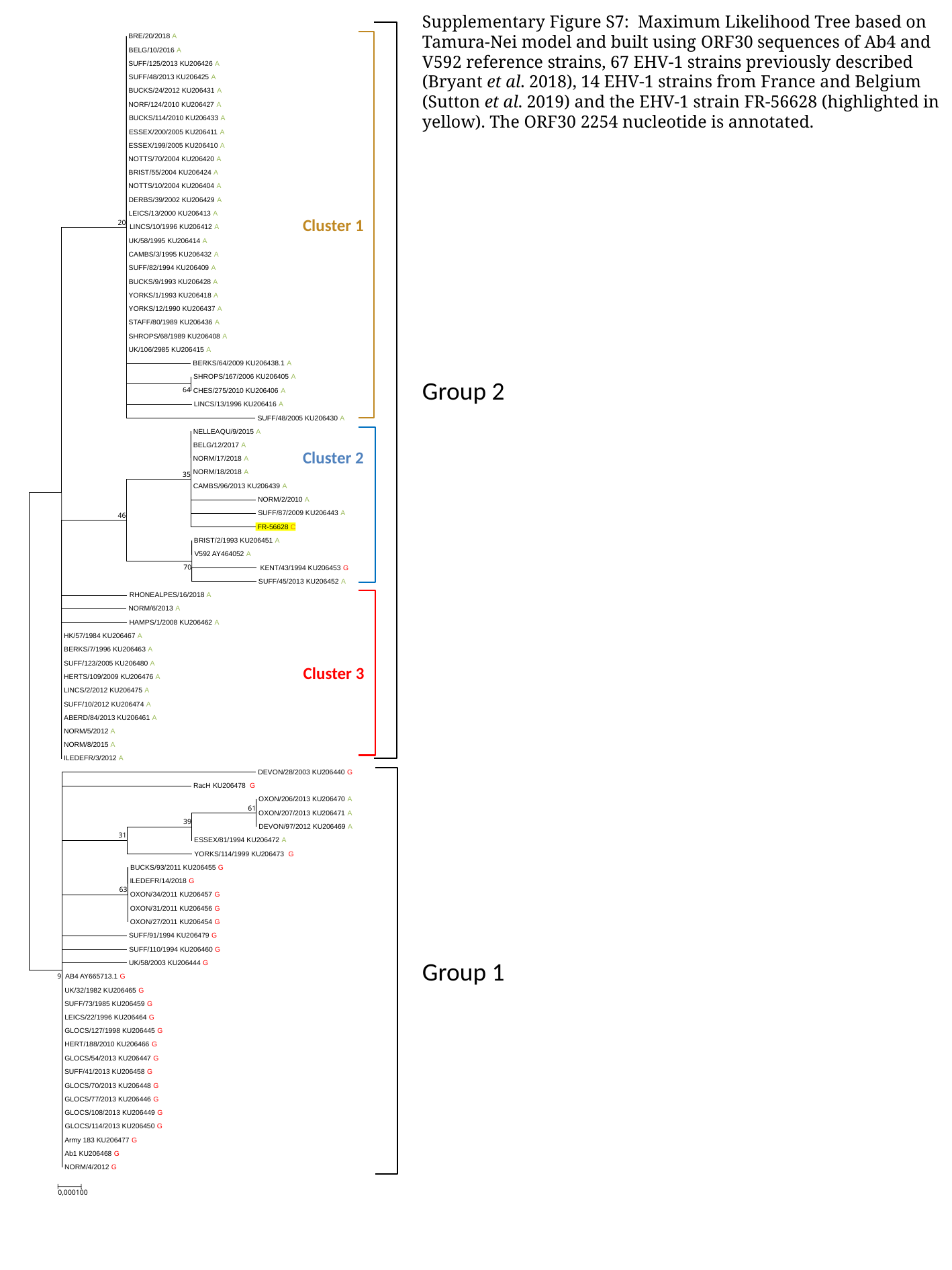

Supplementary Figure S7: Maximum Likelihood Tree based on Tamura-Nei model and built using ORF30 sequences of Ab4 and V592 reference strains, 67 EHV-1 strains previously described (Bryant et al. 2018), 14 EHV-1 strains from France and Belgium (Sutton et al. 2019) and the EHV-1 strain FR-56628 (highlighted in yellow). The ORF30 2254 nucleotide is annotated.
 BRE/20/2018 A
 BELG/10/2016 A
 SUFF/125/2013 KU206426 A
 SUFF/48/2013 KU206425 A
 BUCKS/24/2012 KU206431 A
 NORF/124/2010 KU206427 A
 BUCKS/114/2010 KU206433 A
 ESSEX/200/2005 KU206411 A
 ESSEX/199/2005 KU206410 A
 NOTTS/70/2004 KU206420 A
 BRIST/55/2004 KU206424 A
 NOTTS/10/2004 KU206404 A
 DERBS/39/2002 KU206429 A
 LEICS/13/2000 KU206413 A
20
 LINCS/10/1996 KU206412 A
 UK/58/1995 KU206414 A
 CAMBS/3/1995 KU206432 A
 SUFF/82/1994 KU206409 A
 BUCKS/9/1993 KU206428 A
 YORKS/1/1993 KU206418 A
 YORKS/12/1990 KU206437 A
 STAFF/80/1989 KU206436 A
 SHROPS/68/1989 KU206408 A
 UK/106/2985 KU206415 A
 BERKS/64/2009 KU206438.1 A
 SHROPS/167/2006 KU206405 A
64
 CHES/275/2010 KU206406 A
 LINCS/13/1996 KU206416 A
 SUFF/48/2005 KU206430 A
 NELLEAQU/9/2015 A
 BELG/12/2017 A
 NORM/17/2018 A
 NORM/18/2018 A
35
 CAMBS/96/2013 KU206439 A
 NORM/2/2010 A
 SUFF/87/2009 KU206443 A
46
 FR-56628 C
 BRIST/2/1993 KU206451 A
 V592 AY464052 A
70
 KENT/43/1994 KU206453 G
 SUFF/45/2013 KU206452 A
 RHONEALPES/16/2018 A
 NORM/6/2013 A
 HAMPS/1/2008 KU206462 A
 HK/57/1984 KU206467 A
 BERKS/7/1996 KU206463 A
 SUFF/123/2005 KU206480 A
 HERTS/109/2009 KU206476 A
 LINCS/2/2012 KU206475 A
 SUFF/10/2012 KU206474 A
 ABERD/84/2013 KU206461 A
 NORM/5/2012 A
 NORM/8/2015 A
 ILEDEFR/3/2012 A
 DEVON/28/2003 KU206440 G
 RacH KU206478 G
 OXON/206/2013 KU206470 A
61
 OXON/207/2013 KU206471 A
39
 DEVON/97/2012 KU206469 A
31
 ESSEX/81/1994 KU206472 A
 YORKS/114/1999 KU206473 G
 BUCKS/93/2011 KU206455 G
 ILEDEFR/14/2018 G
63
 OXON/34/2011 KU206457 G
 OXON/31/2011 KU206456 G
 OXON/27/2011 KU206454 G
 SUFF/91/1994 KU206479 G
 SUFF/110/1994 KU206460 G
 UK/58/2003 KU206444 G
9
 AB4 AY665713.1 G
 UK/32/1982 KU206465 G
 SUFF/73/1985 KU206459 G
 LEICS/22/1996 KU206464 G
 GLOCS/127/1998 KU206445 G
 HERT/188/2010 KU206466 G
 GLOCS/54/2013 KU206447 G
 SUFF/41/2013 KU206458 G
 GLOCS/70/2013 KU206448 G
 GLOCS/77/2013 KU206446 G
 GLOCS/108/2013 KU206449 G
 GLOCS/114/2013 KU206450 G
 Army 183 KU206477 G
 Ab1 KU206468 G
 NORM/4/2012 G
0,000100
Cluster 1
Group 2
Cluster 2
Cluster 3
Group 1

## Slide 15
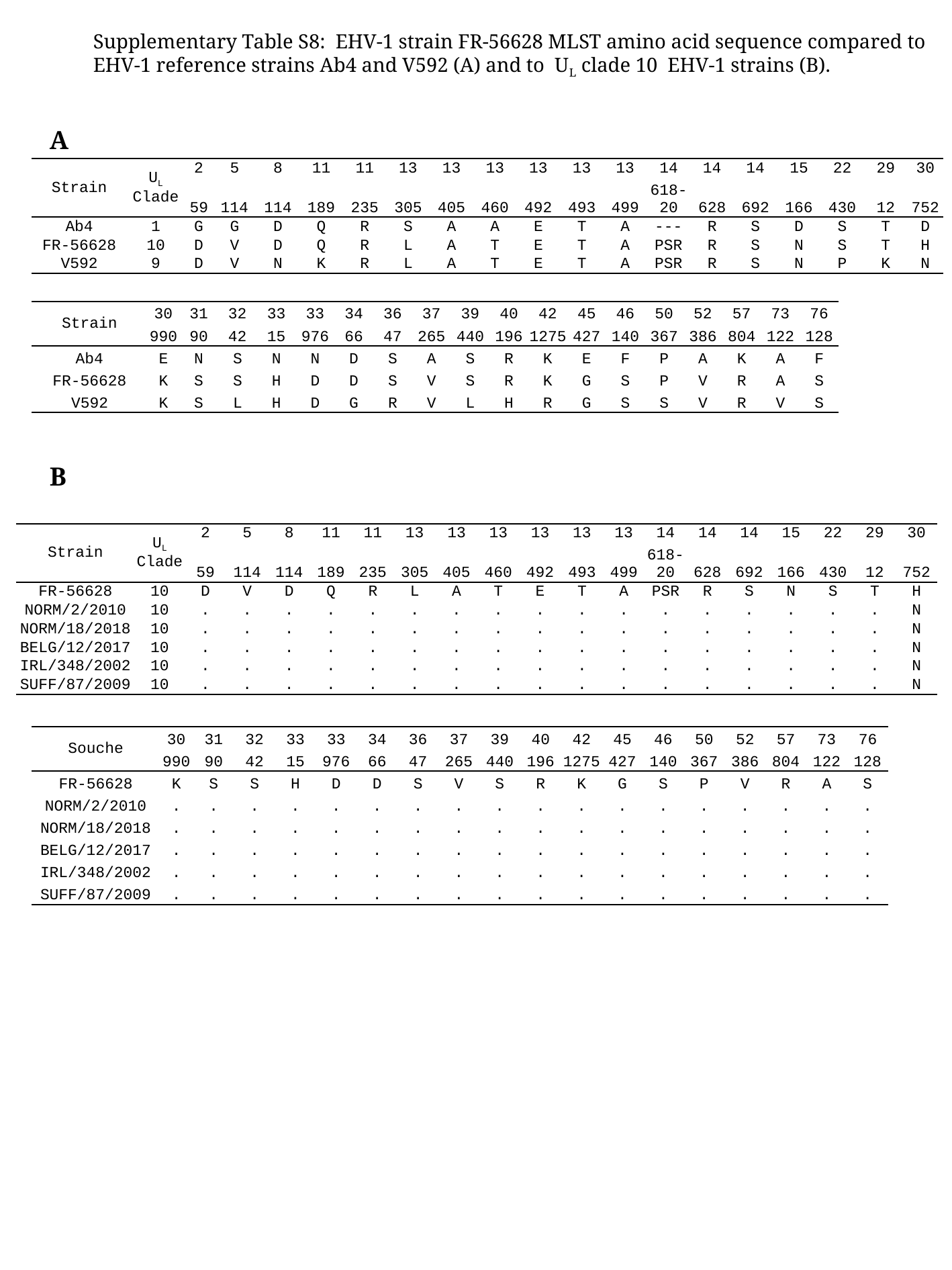

Supplementary Table S8: EHV-1 strain FR-56628 MLST amino acid sequence compared to EHV-1 reference strains Ab4 and V592 (A) and to UL clade 10 EHV-1 strains (B).
A
| Strain | UL Clade | 2 | 5 | 8 | 11 | 11 | 13 | 13 | 13 | 13 | 13 | 13 | 14 | 14 | 14 | 15 | 22 | 29 | 30 |
| --- | --- | --- | --- | --- | --- | --- | --- | --- | --- | --- | --- | --- | --- | --- | --- | --- | --- | --- | --- |
| | | 59 | 114 | 114 | 189 | 235 | 305 | 405 | 460 | 492 | 493 | 499 | 618-20 | 628 | 692 | 166 | 430 | 12 | 752 |
| Ab4 | 1 | G | G | D | Q | R | S | A | A | E | T | A | --- | R | S | D | S | T | D |
| FR-56628 | 10 | D | V | D | Q | R | L | A | T | E | T | A | PSR | R | S | N | S | T | H |
| V592 | 9 | D | V | N | K | R | L | A | T | E | T | A | PSR | R | S | N | P | K | N |
| Strain | 30 | 31 | 32 | 33 | 33 | 34 | 36 | 37 | 39 | 40 | 42 | 45 | 46 | 50 | 52 | 57 | 73 | 76 |
| --- | --- | --- | --- | --- | --- | --- | --- | --- | --- | --- | --- | --- | --- | --- | --- | --- | --- | --- |
| | 990 | 90 | 42 | 15 | 976 | 66 | 47 | 265 | 440 | 196 | 1275 | 427 | 140 | 367 | 386 | 804 | 122 | 128 |
| Ab4 | E | N | S | N | N | D | S | A | S | R | K | E | F | P | A | K | A | F |
| FR-56628 | K | S | S | H | D | D | S | V | S | R | K | G | S | P | V | R | A | S |
| V592 | K | S | L | H | D | G | R | V | L | H | R | G | S | S | V | R | V | S |
B
| Strain | UL Clade | 2 | 5 | 8 | 11 | 11 | 13 | 13 | 13 | 13 | 13 | 13 | 14 | 14 | 14 | 15 | 22 | 29 | 30 |
| --- | --- | --- | --- | --- | --- | --- | --- | --- | --- | --- | --- | --- | --- | --- | --- | --- | --- | --- | --- |
| | | 59 | 114 | 114 | 189 | 235 | 305 | 405 | 460 | 492 | 493 | 499 | 618-20 | 628 | 692 | 166 | 430 | 12 | 752 |
| FR-56628 | 10 | D | V | D | Q | R | L | A | T | E | T | A | PSR | R | S | N | S | T | H |
| NORM/2/2010 | 10 | . | . | . | . | . | . | . | . | . | . | . | . | . | . | . | . | . | N |
| NORM/18/2018 | 10 | . | . | . | . | . | . | . | . | . | . | . | . | . | . | . | . | . | N |
| BELG/12/2017 | 10 | . | . | . | . | . | . | . | . | . | . | . | . | . | . | . | . | . | N |
| IRL/348/2002 | 10 | . | . | . | . | . | . | . | . | . | . | . | . | . | . | . | . | . | N |
| SUFF/87/2009 | 10 | . | . | . | . | . | . | . | . | . | . | . | . | . | . | . | . | . | N |
| Souche | 30 | 31 | 32 | 33 | 33 | 34 | 36 | 37 | 39 | 40 | 42 | 45 | 46 | 50 | 52 | 57 | 73 | 76 |
| --- | --- | --- | --- | --- | --- | --- | --- | --- | --- | --- | --- | --- | --- | --- | --- | --- | --- | --- |
| | 990 | 90 | 42 | 15 | 976 | 66 | 47 | 265 | 440 | 196 | 1275 | 427 | 140 | 367 | 386 | 804 | 122 | 128 |
| FR-56628 | K | S | S | H | D | D | S | V | S | R | K | G | S | P | V | R | A | S |
| NORM/2/2010 | . | . | . | . | . | . | . | . | . | . | . | . | . | . | . | . | . | . |
| NORM/18/2018 | . | . | . | . | . | . | . | . | . | . | . | . | . | . | . | . | . | . |
| BELG/12/2017 | . | . | . | . | . | . | . | . | . | . | . | . | . | . | . | . | . | . |
| IRL/348/2002 | . | . | . | . | . | . | . | . | . | . | . | . | . | . | . | . | . | . |
| SUFF/87/2009 | . | . | . | . | . | . | . | . | . | . | . | . | . | . | . | . | . | . |

## Slide 16
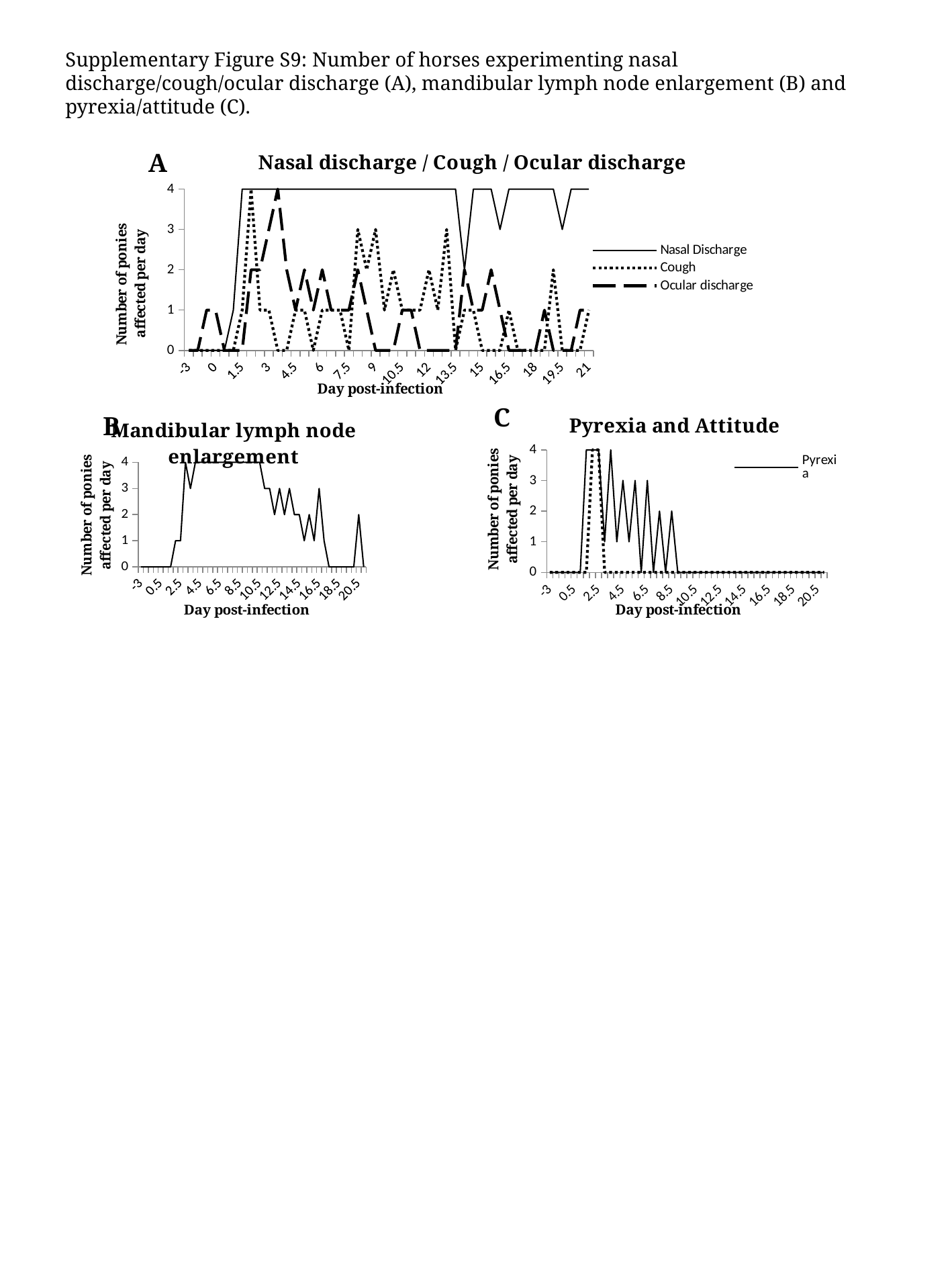

Supplementary Figure S9: Number of horses experimenting nasal discharge/cough/ocular discharge (A), mandibular lymph node enlargement (B) and pyrexia/attitude (C).
### Chart: Nasal discharge / Cough / Ocular discharge
| Category | Nasal Discharge | Cough | Ocular discharge |
|---|---|---|---|
| -3 | 0.0 | 0.0 | 0.0 |
| -2 | 0.0 | 0.0 | 0.0 |
| -1 | 0.0 | 0.0 | 1.0 |
| 0 | 0.0 | 0.0 | 1.0 |
| 0.5 | 0.0 | 0.0 | 0.0 |
| 1 | 1.0 | 0.0 | 0.0 |
| 1.5 | 4.0 | 1.0 | 0.0 |
| 2 | 4.0 | 4.0 | 2.0 |
| 2.5 | 4.0 | 1.0 | 2.0 |
| 3 | 4.0 | 1.0 | 3.0 |
| 3.5 | 4.0 | 0.0 | 4.0 |
| 4 | 4.0 | 0.0 | 2.0 |
| 4.5 | 4.0 | 1.0 | 1.0 |
| 5 | 4.0 | 1.0 | 2.0 |
| 5.5 | 4.0 | 0.0 | 1.0 |
| 6 | 4.0 | 1.0 | 2.0 |
| 6.5 | 4.0 | 1.0 | 1.0 |
| 7 | 4.0 | 1.0 | 1.0 |
| 7.5 | 4.0 | 0.0 | 1.0 |
| 8 | 4.0 | 3.0 | 2.0 |
| 8.5 | 4.0 | 2.0 | 1.0 |
| 9 | 4.0 | 3.0 | 0.0 |
| 9.5 | 4.0 | 1.0 | 0.0 |
| 10 | 4.0 | 2.0 | 0.0 |
| 10.5 | 4.0 | 1.0 | 1.0 |
| 11 | 4.0 | 1.0 | 1.0 |
| 11.5 | 4.0 | 1.0 | 0.0 |
| 12 | 4.0 | 2.0 | 0.0 |
| 12.5 | 4.0 | 1.0 | 0.0 |
| 13 | 4.0 | 3.0 | 0.0 |
| 13.5 | 4.0 | 0.0 | 0.0 |
| 14 | 2.0 | 1.0 | 2.0 |
| 14.5 | 4.0 | 1.0 | 1.0 |
| 15 | 4.0 | 0.0 | 1.0 |
| 15.5 | 4.0 | 0.0 | 2.0 |
| 16 | 3.0 | 0.0 | 1.0 |
| 16.5 | 4.0 | 1.0 | 0.0 |
| 17 | 4.0 | 0.0 | 0.0 |
| 17.5 | 4.0 | 0.0 | 0.0 |
| 18 | 4.0 | 0.0 | 0.0 |
| 18.5 | 4.0 | 0.0 | 1.0 |
| 19 | 4.0 | 2.0 | 0.0 |
| 19.5 | 3.0 | 0.0 | 0.0 |
| 20 | 4.0 | 0.0 | 0.0 |
| 20.5 | 4.0 | 0.0 | 1.0 |
| 21 | 4.0 | 1.0 | 1.0 |A
C
### Chart:
| Category | Mandibular lymph node enlargement |
|---|---|
| -3 | 0.0 |
| -2 | 0.0 |
| -1 | 0.0 |
| 0 | 0.0 |
| 0.5 | 0.0 |
| 1 | 0.0 |
| 1.5 | 0.0 |
| 2 | 1.0 |
| 2.5 | 1.0 |
| 3 | 4.0 |
| 3.5 | 3.0 |
| 4 | 4.0 |
| 4.5 | 4.0 |
| 5 | 4.0 |
| 5.5 | 4.0 |
| 6 | 4.0 |
| 6.5 | 4.0 |
| 7 | 4.0 |
| 7.5 | 4.0 |
| 8 | 4.0 |
| 8.5 | 4.0 |
| 9 | 4.0 |
| 9.5 | 4.0 |
| 10 | 4.0 |
| 10.5 | 4.0 |
| 11 | 3.0 |
| 11.5 | 3.0 |
| 12 | 2.0 |
| 12.5 | 3.0 |
| 13 | 2.0 |
| 13.5 | 3.0 |
| 14 | 2.0 |
| 14.5 | 2.0 |
| 15 | 1.0 |
| 15.5 | 2.0 |
| 16 | 1.0 |
| 16.5 | 3.0 |
| 17 | 1.0 |
| 17.5 | 0.0 |
| 18 | 0.0 |
| 18.5 | 0.0 |
| 19 | 0.0 |
| 19.5 | 0.0 |
| 20 | 0.0 |
| 20.5 | 2.0 |
| 21 | 0.0 |B
### Chart: Pyrexia and Attitude
| Category | Pyrexia | Attitude |
|---|---|---|
| -3 | 0.0 | 0.0 |
| -2 | 0.0 | 0.0 |
| -1 | 0.0 | 0.0 |
| 0 | 0.0 | 0.0 |
| 0.5 | 0.0 | 0.0 |
| 1 | 0.0 | 0.0 |
| 1.5 | 4.0 | 0.0 |
| 2 | 4.0 | 4.0 |
| 2.5 | 4.0 | 4.0 |
| 3 | 1.0 | 0.0 |
| 3.5 | 4.0 | 0.0 |
| 4 | 1.0 | 0.0 |
| 4.5 | 3.0 | 0.0 |
| 5 | 1.0 | 0.0 |
| 5.5 | 3.0 | 0.0 |
| 6 | 0.0 | 0.0 |
| 6.5 | 3.0 | 0.0 |
| 7 | 0.0 | 0.0 |
| 7.5 | 2.0 | 0.0 |
| 8 | 0.0 | 0.0 |
| 8.5 | 2.0 | 0.0 |
| 9 | 0.0 | 0.0 |
| 9.5 | 0.0 | 0.0 |
| 10 | 0.0 | 0.0 |
| 10.5 | 0.0 | 0.0 |
| 11 | 0.0 | 0.0 |
| 11.5 | 0.0 | 0.0 |
| 12 | 0.0 | 0.0 |
| 12.5 | 0.0 | 0.0 |
| 13 | 0.0 | 0.0 |
| 13.5 | 0.0 | 0.0 |
| 14 | 0.0 | 0.0 |
| 14.5 | 0.0 | 0.0 |
| 15 | 0.0 | 0.0 |
| 15.5 | 0.0 | 0.0 |
| 16 | 0.0 | 0.0 |
| 16.5 | 0.0 | 0.0 |
| 17 | 0.0 | 0.0 |
| 17.5 | 0.0 | 0.0 |
| 18 | 0.0 | 0.0 |
| 18.5 | 0.0 | 0.0 |
| 19 | 0.0 | 0.0 |
| 19.5 | 0.0 | 0.0 |
| 20 | 0.0 | 0.0 |
| 20.5 | 0.0 | 0.0 |
| 21 | 0.0 | 0.0 |

## Slide 17
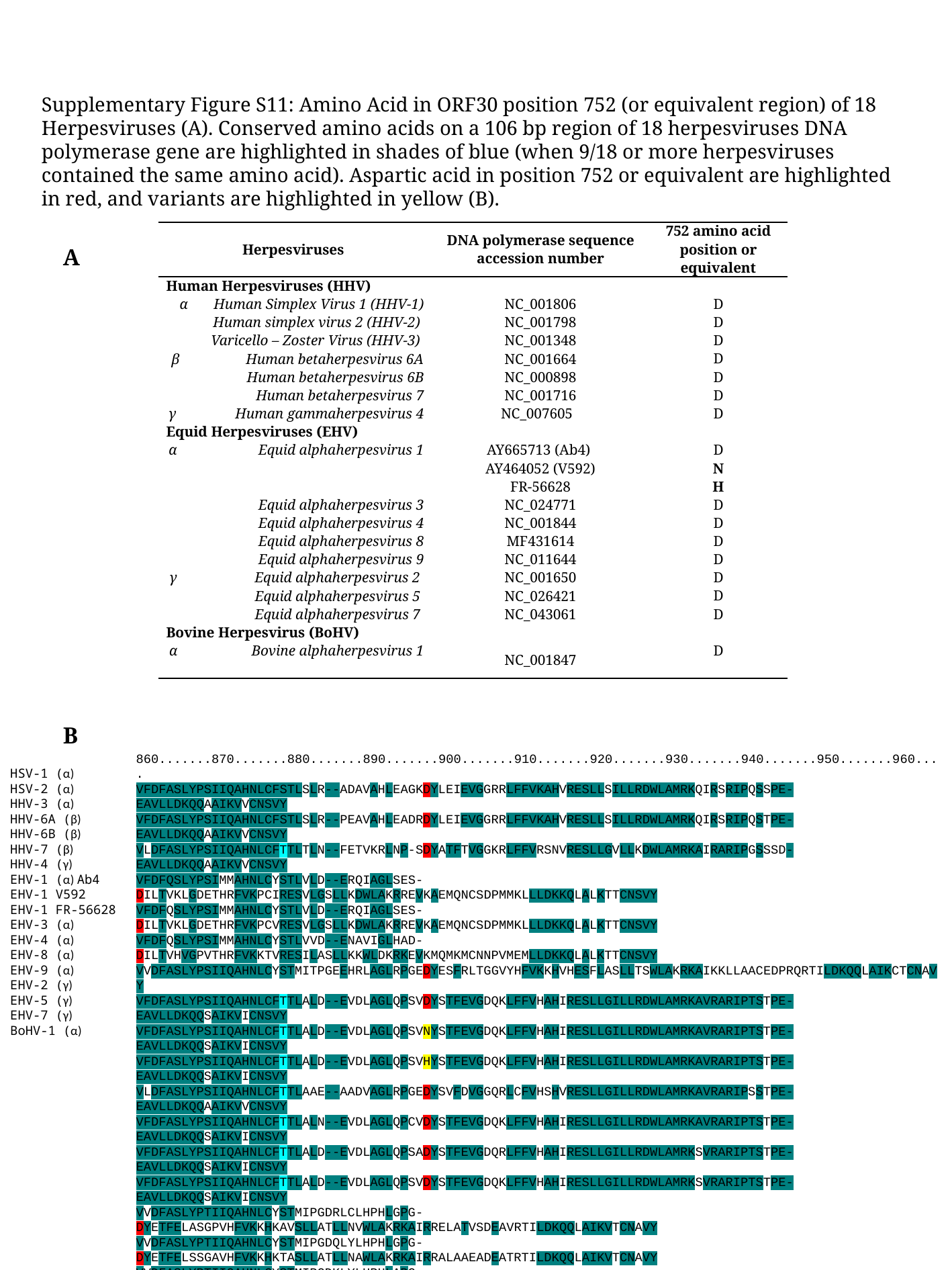

Supplementary Figure S11: Amino Acid in ORF30 position 752 (or equivalent region) of 18 Herpesviruses (A). Conserved amino acids on a 106 bp region of 18 herpesviruses DNA polymerase gene are highlighted in shades of blue (when 9/18 or more herpesviruses contained the same amino acid). Aspartic acid in position 752 or equivalent are highlighted in red, and variants are highlighted in yellow (B).
| Herpesviruses | DNA polymerase sequence accession number | 752 amino acid position or equivalent |
| --- | --- | --- |
| Human Herpesviruses (HHV) | | |
| α Human Simplex Virus 1 (HHV-1) | NC\_001806 | D |
| Human simplex virus 2 (HHV-2) | NC\_001798 | D |
| Varicello – Zoster Virus (HHV-3) | NC\_001348 | D |
| β Human betaherpesvirus 6A | NC\_001664 | D |
| Human betaherpesvirus 6B | NC\_000898 | D |
| Human betaherpesvirus 7 | NC\_001716 | D |
| γ Human gammaherpesvirus 4 | NC\_007605 | D |
| Equid Herpesviruses (EHV) | | |
| α Equid alphaherpesvirus 1 | AY665713 (Ab4) | D |
| | AY464052 (V592) | N |
| | FR-56628 | H |
| Equid alphaherpesvirus 3 | NC\_024771 | D |
| Equid alphaherpesvirus 4 | NC\_001844 | D |
| Equid alphaherpesvirus 8 | MF431614 | D |
| Equid alphaherpesvirus 9 | NC\_011644 | D |
| γ Equid alphaherpesvirus 2 | NC\_001650 | D |
| Equid alphaherpesvirus 5 | NC\_026421 | D |
| Equid alphaherpesvirus 7 | NC\_043061 | D |
| Bovine Herpesvirus (BoHV) | | |
| α Bovine alphaherpesvirus 1 | NC\_001847 | D |
A
B
860.......870.......880.......890.......900.......910.......920.......930.......940.......950.......960....
VFDFASLYPSIIQAHNLCFSTLSLR--ADAVAHLEAGKDYLEIEVGGRRLFFVKAHVRESLLSILLRDWLAMRKQIRSRIPQSSPE-EAVLLDKQQAAIKVVCNSVY
VFDFASLYPSIIQAHNLCFSTLSLR--PEAVAHLEADRDYLEIEVGGRRLFFVKAHVRESLLSILLRDWLAMRKQIRSRIPQSTPE-EAVLLDKQQAAIKVVCNSVY
VLDFASLYPSIIQAHNLCFTTLTLN--FETVKRLNP-SDYATFTVGGKRLFFVRSNVRESLLGVLLKDWLAMRKAIRARIPGSSSD-EAVLLDKQQAAIKVVCNSVY
VFDFQSLYPSIMMAHNLCYSTLVLD--ERQIAGLSES-DILTVKLGDETHRFVKPCIRESVLGSLLKDWLAKRREVKAEMQNCSDPMMKLLLDKKQLALKTTCNSVY
VFDFQSLYPSIMMAHNLCYSTLVLD--ERQIAGLSES-DILTVKLGDETHRFVKPCVRESVLGSLLKDWLAKRREVKAEMQNCSDPMMKLLLDKKQLALKTTCNSVY
VFDFQSLYPSIMMAHNLCYSTLVVD--ENAVIGLHAD-DILTVHVGPVTHRFVKKTVRESILASLLKKWLDKRKEVKMQMKMCNNPVMEMLLDKKQLALKTTCNSVY
VVDFASLYPSIIQAHNLCYSTMITPGEEHRLAGLRPGEDYESFRLTGGVYHFVKKHVHESFLASLLTSWLAKRKAIKKLLAACEDPRQRTILDKQQLAIKCTCNAVY
VFDFASLYPSIIQAHNLCFTTLALD--EVDLAGLQPSVDYSTFEVGDQKLFFVHAHIRESLLGILLRDWLAMRKAVRARIPTSTPE-EAVLLDKQQSAIKVICNSVY
VFDFASLYPSIIQAHNLCFTTLALD--EVDLAGLQPSVNYSTFEVGDQKLFFVHAHIRESLLGILLRDWLAMRKAVRARIPTSTPE-EAVLLDKQQSAIKVICNSVY
VFDFASLYPSIIQAHNLCFTTLALD--EVDLAGLQPSVHYSTFEVGDQKLFFVHAHIRESLLGILLRDWLAMRKAVRARIPTSTPE-EAVLLDKQQSAIKVICNSVY
VLDFASLYPSIIQAHNLCFTTLAAE--AADVAGLRPGEDYSVFDVGGQRLCFVHSHVRESLLGILLRDWLAMRKAVRARIPSSTPE-EAVLLDKQQAAIKVVCNSVY
VFDFASLYPSIIQAHNLCFTTLALN--EVDLAGLQPCVDYSTFEVGDQKLFFVHAHIRESLLGILLRDWLAMRKAVRARIPTSTPE-EAVLLDKQQSAIKVICNSVY
VFDFASLYPSIIQAHNLCFTTLALD--EVDLAGLQPSADYSTFEVGDQRLFFVHAHIRESLLGILLRDWLAMRKSVRARIPTSTPE-EAVLLDKQQSAIKVICNSVY
VFDFASLYPSIIQAHNLCFTTLALD--EVDLAGLQPSVDYSTFEVGDQKLFFVHAHIRESLLGILLRDWLAMRKSVRARIPTSTPE-EAVLLDKQQSAIKVICNSVY
VVDFASLYPTIIQAHNLCYSTMIPGDRLCLHPHLGPG-DYETFELASGPVHFVKKHKAVSLLATLLNVWLAKRKAIRRELATVSDEAVRTILDKQQLAIKVTCNAVY
VVDFASLYPTIIQAHNLCYSTMIPGDQLYLHPHLGPG-DYETFELSSGAVHFVKKHKTASLLATLLNAWLAKRKAIRRALAAEADEATRTILDKQQLAIKVTCNAVY
VVDFASLYPTIIQAHNLCYSTMIPGDKLYLHPHLAPG-DYETFDLSSGTVHFVKKHKTVSLLATLLNVWLAKRKAIRKTLATEADEATRTILDKQQLAIKVTCNAVY
VLDFASLYPSIIQAHNLCFTTLVRR--EAAPAGLTPGADYATFDVGGRALHFVRAHVRESLLSVLLRDWLAMRKAIRARIPTAAPE-EAVLLDKQQAAIKVVCNSVY
HSV-1 (α)
HSV-2 (α)
HHV-3 (α)
HHV-6A (β)
HHV-6B (β)
HHV-7 (β)
HHV-4 (γ)
EHV-1 (α) Ab4
EHV-1 V592
EHV-1 FR-56628
EHV-3 (α)
EHV-4 (α)
EHV-8 (α)
EHV-9 (α)
EHV-2 (γ)
EHV-5 (γ)
EHV-7 (γ)
BoHV-1 (α)
