# Supplementary material for: Identification of a New Equid Herpesvirus 1 DNA Polymerase (ORF30) Genotype with the Isolation of a C2254/H752 Strain in French Horses Showing no Major Impact on the Strain Behaviour
Source: Viruses. 2020 Oct 13;12(10):1160. doi: 10.3390/v12101160 (PMC7650556; doi:10.3390/v12101160)
Supplement: Supplementary file 1 [file viruses-12-01160-s001.zip › Sutton et al 2020 EHV-1 Viruses Supplementary Material-2 11-10-2020.pptx]

## Slide 1
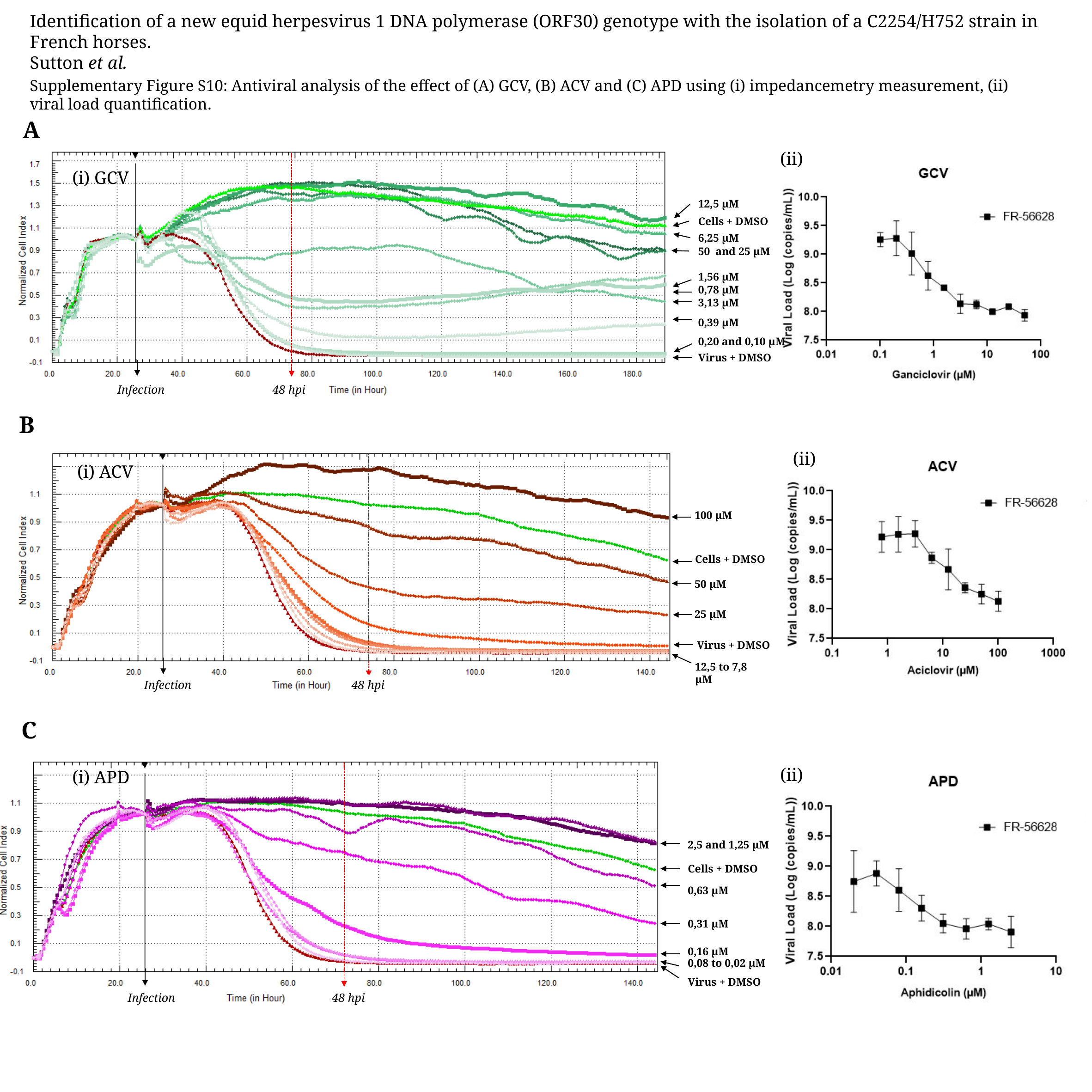

Identification of a new equid herpesvirus 1 DNA polymerase (ORF30) genotype with the isolation of a C2254/H752 strain in French horses.
Sutton et al.
Supplementary Figure S10: Antiviral analysis of the effect of (A) GCV, (B) ACV and (C) APD using (i) impedancemetry measurement, (ii) viral load quantification.
A
(ii)
(i) GCV
12,5 µM
Cells + DMSO
6,25 µM
50 and 25 µM
1,56 µM
0,78 µM
3,13 µM
0,39 µM
0,20 and 0,10 µM
Virus + DMSO
Infection
48 hpi
B
(ii)
(i) ACV
100 µM
Cells + DMSO
50 µM
25 µM
Virus + DMSO
12,5 to 7,8 µM
Infection
 48 hpi
C
(ii)
(i) APD
2,5 and 1,25 µM
Cells + DMSO
0,63 µM
0,31 µM
0,16 µM
0,08 to 0,02 µM
Virus + DMSO
Infection
 48 hpi
